# Energy Decomposition Analysis of the Activation of CO_2_ by Frustrated Lewis Pairs

**DOI:** 10.1021/acs.jpca.5c06202

**Published:** 2025-10-23

**Authors:** Manoj Wijesingha, Xinru Peng, Nathan Hoang, Nicholas Jamieson, Sherine O. Obare, Yirong Mo

**Affiliations:** Department of Nanoscience, Joint School of Nanoscience & Nanoengineering, 14616 University of North Carolina at Greensboro, Greensboro, North Carolina 27401, United States

## Abstract

Numerous frustrated Lewis pairs (FLPs) have been developed for activating small molecules such as H_2_ and CO_2_ in metal-free catalysis. In this work, we applied our energy decomposition scheme based on the block-localized wave function (BLW) method to explore the governing forces in the CO_2_ activations by a series of intramolecular FLPs where both the Lewis acid (with B as the center) and Lewis base (with N or P as the center) are attached to a benzene ring. The uniqueness of the BLW method is the self-consistent derivation of the diabatic state where the charge transfer interactions between FLPs and CO_2_ are completely quenched, allowing us to evaluate the impact of the charge transfer on both geometries and energetics. We showed that the structural rearrangements prepared for the activated states cost considerable energy, leading to the small overall binding energies, which are often even positive (destabilizing). Thus, one key suggestion for the rational design of FLPs is the fixation of the Lewis acid and base centers, together with the substituent groups bonding to these centers. All FLPs studied in this work exhibit strong chemical interactions with CO_2_ in the activated states by donating electrons from FLPs to CO_2_, leading to the activation of CO_2_ with a bent geometry. Approximate linear correlation between the charge transfer energies and the activated CO bond lengths in CO_2_ is observed. Without the charge transfer, FLPs would be unable to absorb and activate CO_2_ with their intramolecular electric fields alone.

## Graphical abstract

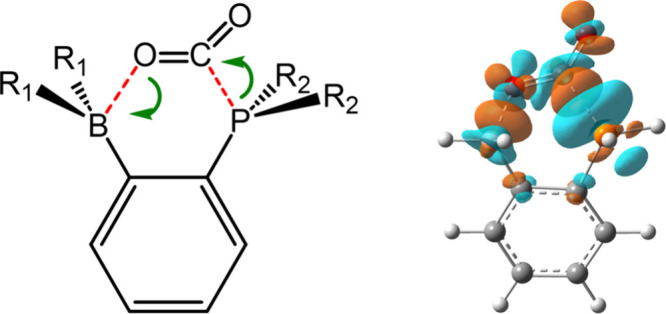

## Introduction

Currently, most chemical reactions require the assistance of catalysts, and most important catalysts are composed of precious transition metals such as Pd and Pt. While these precious transition metals have good catalytic performance in general, the scarcity, high cost, toxicity, and environmental issues associated with them impose challenges for researchers to find alternative solutions using earth-abundant and non-noble metals and main-group elements. Thus, there are significant emerging interests in the development of catalysts free of noble metals, or even completely metal-free.^1–4^ Among the catalysts based on earth-abundant transition metals and main-group elements, one type of compound called frustrated Lewis pairs (FLPs) stands out due to their superior performance in homogeneous catalysis for the activation of a range of small molecules.^5–9^ FLPs, first reported by the Stephan group in 2006,^10^ are a combination of Lewis acids and bases that are sterically or geometrically precluded from forming Lewis acid–base adducts.^11–13^ The advantages of FLPs lie in their versatility of possible structures and catalytic properties, making them potential candidates to replace precious metal-based catalysts in green chemical processes.^14,15^ Typical examples of FLPs are inter- or intramolecular combinations of bulky bases such as phosphines or amines (*t*-Bu_3_P, Mes_3_P, and amines) with strongly electrophilic components such as B­(C_6_F_5_)_3_. Rigid intramolecular FLPs have been built on scaffolds (xanthene, adamantane, etc.) that fix the distance between the basic and acidic centers (e.g., the P···B distance). These scaffolds not only lower strain but also improve reactivity.^16,17^ Recently, based on the synergistic effect of Lewis acid and base, a series of FLPs such as Al/P, B/C, B/N, P/N, and B/P or even Si^+^/N and Ge^+^/N have been developed in the homogeneous catalysis and heterogeneous systems for the hydrogenation of unsaturated molecules such as C_2_H_2_ and CO_2_ ^10,13,18–30^ and the heterolytic splitting of H_2_.^31–33^ Other FLP systems include N-heterocyclic carbenes as the Lewis base and a cationic Si or Ge center as the Lewis acid, where N­(lp) → CO_2_(π*) donation and O­(lp) → Si acceptor interactions rule the activation of CO_2_.^34,35^ The diversity of FLP systems greatly enriches the study of CO_2_ reduction.^35–40^ To increase the efficiency and selectivity of FLPs in catalysis, however, it is essential to identify the electronic and structural factors that are capable of fine-tuning the chemisorption and subsequent reactivity and stability of these FLP systems.

We are concerned with the continuous increase in the global atmospheric level of greenhouse gas CO_2_, which results in serious consequences in climate change and human health. To mitigate CO_2_ and reach the goal of zero emission of CO_2_, there are two general strategies, capturing CO_2_ and sequestering it deep in the ground or preferably converting it to value-added commodity chemicals. The latter is advantageous both from a scientific and an environmental perspective.^41–47^ However, CO_2_ is a highly thermodynamically stable molecule with a nonpolar linear structure and is often mixed in low concentration with other stable and nonpolar gases such as N_2_. Therefore, extra energy in the form of electricity or heat is required whether to capture CO_2_ through physisorption and sequester it or through chemical adsorption and conversion.^37^ We aim to improve the efficiency of these processes by a fundamental understanding of the molecular interactions between CO_2_ and FLPs. Note that often, neither a Lewis base like *t*Bu_3_P nor a Lewis acid like B­(C_6_F_5_)_3_ is independently effective at CO_2_ capture and only their combination in the form of FLP is.^46^ The synergistic interactions of both Lewis base *t*Bu_3_P and Lewis acid B­(C_6_F_5_)_3_ with CO_2_ can lead to the activation of CO_2_, as evidenced by the bent structure of CO_2_ and stretched C=O bond distance.^48^ The general mechanism for the CO_2_ activation by FLPs can be illustrated by Figure 1a, where Lewis base (LB) and acid (LA) can be separated molecules or linked by methylene or ethylene groups as spacers. The chemisorption and activation mainly result from the nucleophilic attraction of C to LB, where the electron transfer from LB to CO_2_ is the key. The attraction between one oxygen atom of CO_2_ and LA is largely supportive. Thus, CO_2_ activation is characterized by the bifunctional interaction between FLPs and CO_2_. For instances, Mes_2_P­(CH_2_)_2_B­(C_6_F_5_)_2_ ^49^ and a P/N-based FLP amidophosphorane can capture CO_2_ at ambient conditions,^50^ and B/N FLPs can mediate the conversion of CO_2_ and H_2_ to generate formyl, diacetal, and methoxyboranes.^51^

**Figure 1:**
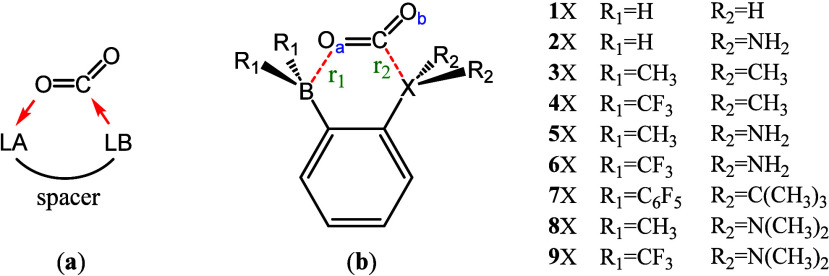
(a) CO_2_ activation by FLPs with red arrows showing the directions of electron transfers; (b) model FLPs studied in the work (X = P and N).

In this work, we theoretically studied a series of intramolecular FLPs where both LA and LB are attached to a distinct backbone, i.e., the benzene ring (Figure 1b), which plays the role of a rigid spacer. By adopting various substituent groups, including electron-donating groups and electron-withdrawing groups at B and N/P, and performing energy decomposition analysis (EDA) and orbital correlation analysis, we aim to elucidate the role of B and N/P cooperativity, find the best combinations, and identify the governing factors, including steric repulsion and electronic effects of substituents in the activation of CO_2_ by FLPs.

## Methodologies and Computational Details

The activation of CO_2_ involves its structural deformation from linear to bent, and this deformation can cost up to 80 kcal/mol.^52^ The final binding energy, which is an experimental observable, is a combination of all factors, including the deformation cost. Thus, the binding energy itself is a poor and unreliable indicator for the magnitude of activation of CO_2_. For instance, our previous study showed that bidentate β-diketiminate (BDI) compounds (BDI)­Al­(I) and (BDI)­Mg^+^ can absorb CO_2_ with binding energies of −17.6 and −22.9 kcal/mol, respectively.^52^ However, in-depth analyses showed that the former can significantly activate CO_2_ by transferring one electron to CO_2_ whereas the latter cannot with negligible electrons transferred. EDA approaches, which decompose the intra- or intermolecular interaction energy into several physically meaningful components based on electronic and steric effects, have been proven to be effective in understanding the physical forces governing molecular bonding and reactivity.^53–75^

In this work, we employed our developed block-localized wave function (BLW) method,^76–79^ which is the simplest variant of ab initio valence bond (VB) theory.^80–82^ The BLW method combines the advantages of both MO and VB theories and thus is of the MO or DFT computational efficiency. Notably, it can derive the wave function self-consistently for a diabatic state (i.e., Lewis state) where all orbital mixings or interactions are deactivated, and in other words, it can address the impact of orbital interactions or electron transfers on molecular geometry, energetics, and spectral properties. Based on the BLW method, intermolecular binding energy can be decomposed to deformation energy (Δ*E*_def_), frozen energy (Δ*E*_F_), polarization (Δ*E*_pol_), and charge transfer (Δ*E*_CT_).^76,77^ For the instance of a complex AB composed of interacting moieties A and B, the binding energy (Δ*E*_b_) computed at the DFT level corresponds to the energy change from optimal monomers A and B to the optimal complex AB as

$$\Delta E_{b}=E\left(\Psi_{\mathrm{AB}}^{\mathrm{DFT}}\right)-\left[E\left(\Psi_{A}^{\mathrm{DFT}}\right)+E\left(\Psi_{B}^{\mathrm{DFT}}\right)\right]+\mathrm{BSSE}=\Delta E_{\mathrm{def}}+\Delta E_{F}+\Delta E_{\mathrm{pol}}+\Delta E_{\mathrm{CT}}=\Delta E_{\mathrm{def}}+\Delta E_{\mathrm{int}}$$ 1

where $\Psi_{\mathrm{AB}}^{\mathrm{DFT}},\Psi_{A}^{\mathrm{DFT}},\mathrm{and}\;\Psi_{B}^{\mathrm{DFT}}$ are the wave functions of the optimal complex AB and monomers A and B, respectively. BSSE is the basis set superposition error evaluated by the Boys–Bernardi’s counterpoise method.^83^ The key in this approach is the definition of the intermediate (diabatic) state where any charge transfer between A and B is strictly quenched. The initial intermediate state is defined as

$$\Psi_{\mathrm{AB}}^{\mathrm{BLW}\;0}=Â\left\{\Psi_{A}^{0}\Psi_{B}^{0}\right\}$$ 2

where $\Psi_{A}^{0}$ and $\Psi_{B}^{0}$ are the wave functions of deformed monomers A and B, respectively. In other words, the block-localized orbitals (or monomeric electron densities in DFT) are frozen at their individual monomers in eq 2. The energy change from $\Psi_{A}^{\mathrm{DFT}}\;\mathrm{or}\;\Psi_{B}^{\mathrm{DFT}}\;\mathrm{to}\;\Psi_{A}^{0}\;\mathrm{or}\;\Psi_{B}^{0}$ is the deformation energy of A or B. The self-consistent optimization of the BLW state leads to

$$\Psi_{\mathrm{AB}}^{\mathrm{BLW}}=Â\left\{\Psi_{A}^{\mathrm{BLW}}\Psi_{B}^{\mathrm{BLW}}\right\}$$ 3

where the block-localized orbitals are self-consistently optimized (i.e., relaxed or polarized). It should be pointed out that the frozen energy (Δ*E*_F_) includes not only electrostatic energy and Pauli repulsion but also electron correlation energy at the DFT level. Since there is no rigorous way to decompose this frozen energy at the DFT level, we will generally refer to this energy term as steric energy. All geometries were fully optimized without constraints (except in BLW geometry optimizations, where the charge transfer among interacting monomers is suppressed) and with the analytic gradients and Berny algorithms using the GEDIIS method. The convergence criteria for electronic structure calculations were set to the default values in either Gaussian 16 ^84^ or GAMESS.^85^ Vibrational frequency computations were performed to verify the minimum geometries of all species. Partial atomic charges were derived from the natural population analysis (NPA) scheme.^86^

The self-consistent derivation of the intermediate diabatic state (eq 3) implies that the orbital energies of A and B can also be derived. Thus, we can track the evolutions of orbital energies and plot out the “*in situ*” orbital correlation diagram, which is superior to the traditional orbital correlation diagram. Traditional orbital correlation diagrams connect the energy-ordered orbitals (usually frontier orbitals HOMOs and LUMOs) of reactants and products based on the conservation of symmetries and major orbital compositions and have become icons in chemistry and valuable tools in understanding chemical reactions and molecular properties.^87^ However, the orbital energies of interacting species in these correlation diagrams are derived from their separated and isolated states, but orbital energies can be influenced by external fields and the existence of neighboring molecules. The definition of the BLW states (eqs 2 and 3) allows us to put interacting species “physically” together without any “chemical” interactions. In such a way, “*in situ*” orbital correlation diagrams can differentiate the impacts of physical interactions and chemical interactions on orbital energy levels, providing deeper insights into the chemical bonding nature than traditional orbital correlation diagrams.

In this work, all regular DFT geometry optimizations were performed using Gaussian 16 software^84^ with the Minnesota density functional (M06-2X)^88–90^ and the all-electron 6-311+G­(d,p) basis set. Atomic charges were derived with the natural population analysis (NPA).^86^ BLW computations, where the electron transfer between FLPs and CO_2_ is strictly disabled, were done at the same M06-2X/6-311+G­(d,p) theoretical level with GAMESS^85^ to which our BLW code was ported in our laboratories. As the BLW method can strictly localize electrons and critically exhibit the energetic and structural changes without any electron transfer interactions, we expect that the applications of the BLW to the absorption, activation, and conversion of CO_2_ by FLPs can provide unique insights and helpful guidelines for the further rational design of FLPs.^15,35^

## Results and Discussion

### Geometries of Free FLPs

We first optimized the geometries of free FLPs with the major structural parameter *r* ^0^(BX) listed in Table 1. It is interesting to note that the separation between the Lewis acid center B and the Lewis base center X (= N and P) is not only influenced by the steric repulsion among the substituent groups but also implicated by the attractive bonding between the substituent groups and Lewis acid/base centers or even between the substituent groups at the two sides. For instance, there are two minimal geometries for **1** (Figure 2) with **1** as the ground state and **1′** as the secondary minimum. The latter can be regarded as the preparation state for CO_2_ activation. **1**N takes a nearly planar geometry due to the electrostatic attraction between the hydrogen atoms bonding to B and N. The electronegativity differences lead to the positive charge on the H attached to N and the negative charge on the H attached to B. The energy gap between **1**N and **1**N′ is 4.8 kcal/mol. For **1** (X = P), similarly, there are two minimal states with **1**P as the ground state. However, the much higher inversion barrier for PH_3_ than for NH_3_ results in **1**P being far from planarity, though the energy difference between **1**P and **1**P′ is similarly 4.7 kcal/mol. Different from **1**, both **2** and **3** prefer the **1′**-like structures as their ground states. While our initial intention with the addition of −NH_2_ groups to the Lewis base center is to increase the basicity of N or P to reduce the key B–X distances as confirmed by the optimal geometries of **2′** in Figure 2, further studies showed that these are only local minima like **1**′, and the ground state structures are featured with the bonding between N of a substituent group −NH_2_ and B with a bond distance of 1.650–1.657 Å, which is essentially the equilibrium N–B distance in BH_3_NH_3_.^91^ Since **2**X′ structures can be regarded as the preparatory states for the CO_2_ addition to the bound structure as shown in Figure 1b, the high stability of **2**X versus **2**X′ (20.03 and 23.00 kcal/mol for X = N and P, respectively) suggests that there will be a high deformation cost to break the binding of the substituent group −NH_2_ and B. Similar structural abnormalities occur for species **5** and **6** where −NH_2_ groups are attached to the Lewis base center. When R_2_=N­(CH_3_)_2_ is used in FLP complexes **8** and **9**, the N atom in one substituent group still tends to form a dative bonding with the boron atom, though the steric repulsion increases a little bit, as reflected by the slightly longer N···B distance (see Figure 2). For complexes **8** and **9** with phosphorus (X = P), however, the strong dative bonding between B and N atoms effectively weakens and stretches the N–P bonds.

**Figure 2:**
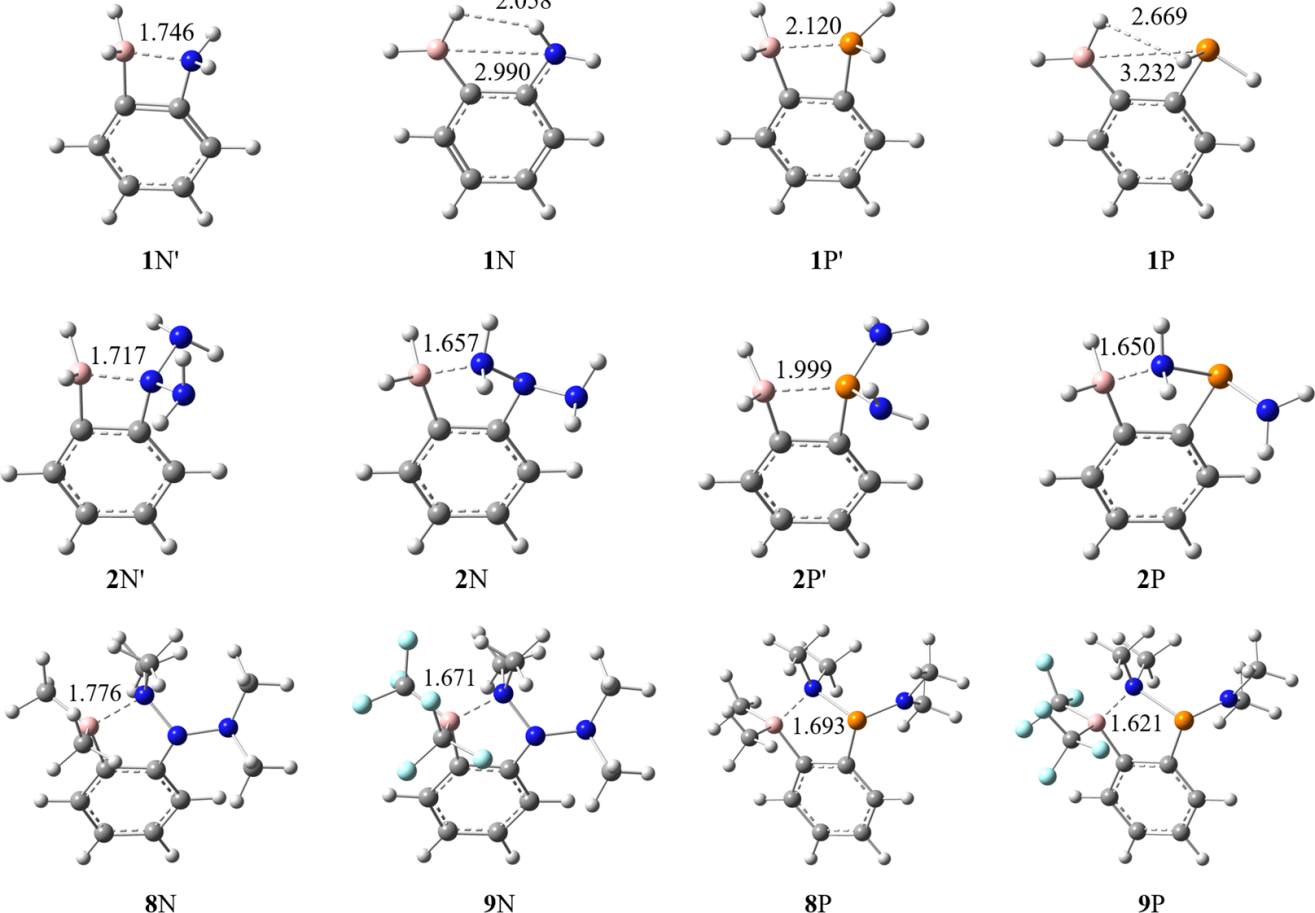
Isomers of complexes **1**, **2**, **8**, and **9**. Bond distances in Å.

**Table 1:** Major Structural Parameters (Bond Lengths in Å, Bond Angles in Degrees) and Natural Atomic Charges (*q*, in e) for FLP and FLP-CO_2_ Complexes

	FLP			FLP-CO_2_					
system	*r* ^0^(BX)	*q*(B)	*q*(X)	*r*(BX)	*r*_1_	*r*_2_	*r*(O_a_C)	*r*(O_b_C)	<O_a_CO_b_
**1**N	2.990	0.422	–0.801	2.669	1.601	1.595	1.250	1.189	135.6
**2**N	2.506	0.069	–0.190	2.845	1.585	1.636	1.248	1.187	134.8
**3**N	1.826	0.747	–0.551	3.001	1.618	1.617	1.249	1.191	133.5
**4**N	1.702	0.456	–0.534	2.952	1.519	1.586	1.270	1.184	130.8
**5**N	2.537	0.596	–0.191	2.974	1.628	1.641	1.239	1.193	134.6
**6**N	2.495	0.340	–0.179	2.938	1.526	1.606	1.259	1.186	131.7
**7**N	3.009	1.007	–0.630	3.049	1.540	1.592	1.275	1.185	128.4
**8**N	2.474	0.756	–0.182	2.985	1.624	1.657	1.240	1.191	134.3
**9**N	2.402	0.469	–0.176	3.007	1.509	1.624	1.263	1.182	130.2
**1**P	3.232	0.458	0.328	2.877	1.584	1.940	1.264	1.195	132.8
**2**P	2.824	0.059	1.161	2.938	1.566	1.890	1.274	1.205	130.0
**3**P	3.127	1.002	0.798	3.147	1.604	1.895	1.264	1.207	130.6
**4**P	2.006	0.021	1.247	3.127	1.516	1.881	1.285	1.199	128.4
**5**P	2.871	0.596	1.158	3.111	1.602	1.891	1.264	1.211	129.9
**6**P	2.862	0.342	1.188	3.116	1.515	1.884	1.283	1.203	127.7
**7**P	2.939	0.959	0.950	3.248	1.526	1.892	1.280	1.204	126.4
**8**P	2.819	0.694	1.240	3.123	1.594	1.898	1.271	1.208	129.1
**9**P	2.824	0.421	1.260	3.138	1.510	1.885	1.292	1.200	127.1

Population analyses of FLPs are supposed to imply the driving forces for the absorption of CO_2_. Indeed, for X = N complexes, the Lewis acid center, or the boron atom, carries positive charges, while the Lewis base center, or the nitrogen atom, carries negative charges. However, the phosphorus atom in all X = P complexes carries positive charges, largely due to its lower electronegativity than carbon. Instead of point charges, we plotted the electrostatic potential (ESP) maps of **1**, **3**, **4**, and **7** in Figure 3. The first impression from the ESP plots is that X = P FLPs exhibit large potential disparity and strengths, indicating stronger electrostatic attractions to CO_2_ than X = N complexes. The second is the interference of the substituent groups, notably the fluorine atoms in **4** and **7**, which show high negative (red) electrostatic potentials, though they are not the reactive sites.

**Figure 3:**
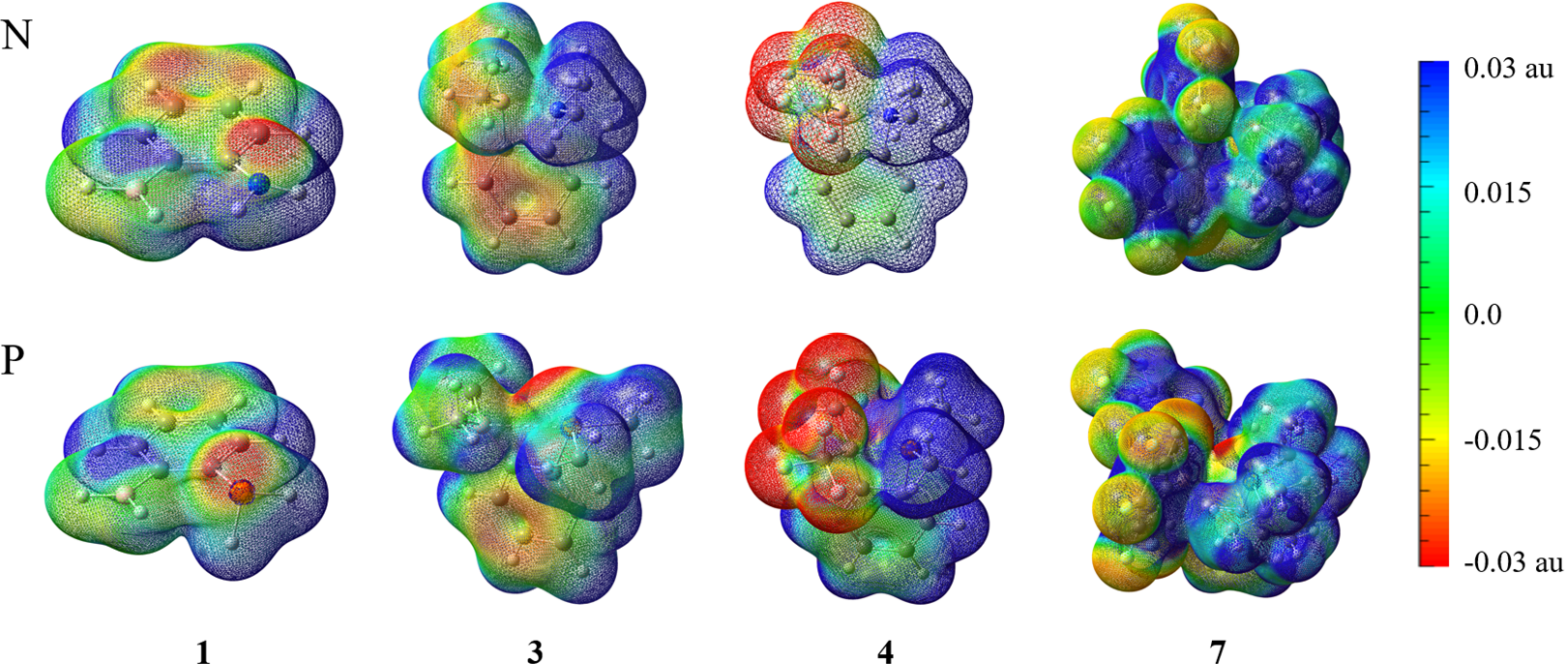
Representative ESP maps showing the negative electrostatic potential site for the binding of CO_2_ by complexes **1**, **3**, **4**, and **7**. Here, ESP is mapped on the 0.005 au electron density isosurfaces, in the range of −0.03 to 0.03 au.

### Geometries of FLPs Bound with CO_2_

With the structures of FLPs optimized, we continued to explore the addition of CO_2_ to these FLPs with the general scheme shown in Figure 1b. Earlier computational studies suggest that the FLP capture of CO_2_ can proceed via a one-step concerted transition state (TS) or a two-step sequence.^49,92^ For instance, Ferrer et al. identified three stationary points in the sequestration of CO_2_ with FLPs based on N-heterocycles with silane/germane groups.^34^ AIMD simulations by Liu et al. showed the capture of CO_2_ by the prototypical *t*-Bu_3_P/B­(C_6_F_5_)_3_ pair via two steps after the entrance of CO_2_ into the cavity of the FLP, which includes the capture of the C of CO_2_ by the Lewis base center P, followed by capture of one O of CO_2_ by the Lewis acid center B.^93^ Our initial geometry optimizations of the parent FLPs 2-borylbenzenamine (**1**N) and (2-borylphenyl)­phosphine (**1**P) showed that the most stable structures with CO_2_ correspond to van der Waals complexes, which result from the electrostatic attraction as implied by the ESP plots in Figure 3. In these van der Waals complexes, CO_2_ is not activated and still exhibits linear geometry with the C=O distance barely changed. As expected, the C and O atoms of CO_2_ are close to X and B, respectively. Adducts of these two FLPs with CO_2_ where CO_2_ is activated (in terms of the bending structure of CO_2_ and the stretching of one C–O bond), are metastable with barriers of 10.75 and 13.26 kcal/mol for X = N and P, respectively, as the energy profiles shown in Figure 4. Since our current major focus is on the nature of CO_2_ activation by FLPs, the following discussion (except the last subsection about the activation barriers) will be based on the FLP-CO_2_ adducts, whose major structural parameters are compiled in Table 1.

**Figure 4:**
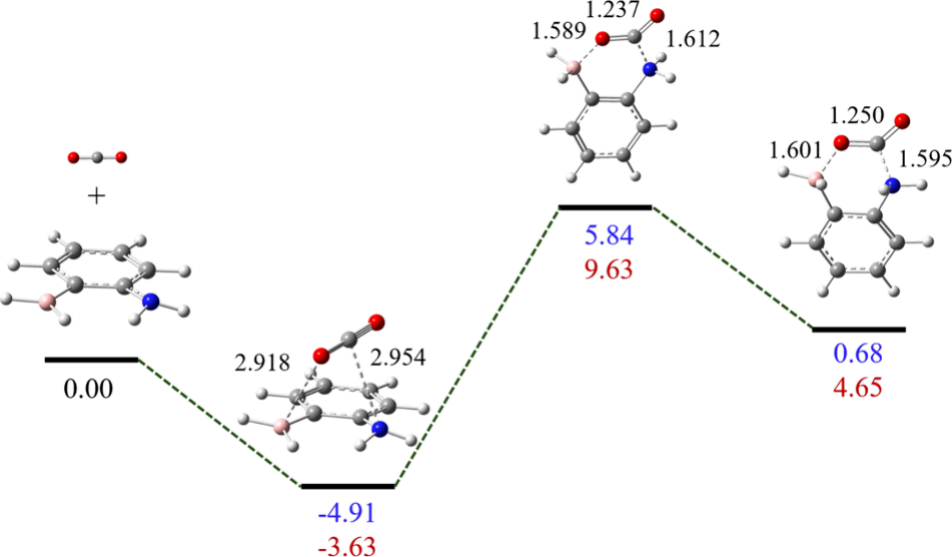
Binding process of complex **1**X (X = N and P) and CO_2_ with structural and relative energy changes (X = N in blue and X = P in brown, energies in kcal/mol and bond distances in Å), computed at the M06-2X/6-311+G­(d,p) level with Gaussian 16 (without the BSSE corrections). Atoms C, O, N, B, and H are represented with balls in gray, red, blue, pink, and white, respectively.

One obvious observation from Table 1 is the elongation of the XB distance with the insertion of CO_2_, which will lead to a high deformation cost (Δ*E*_def_), as reflected by the data in Table 2. On average, the deformation costs for CO_2_ and FLP are comparable. While the deformation of CO_2_ is a part of the activation process and thus is inevitable, the cost for FLP can be minimized by rational designs, e.g., by fixing both Lewis acid and base on a surface. Rigid scaffolds such as surface FLPs can be constructed by taking advantage of surface defects and doping in metal oxides and exhibit high catalytic reactivity.^94–97^ Delarmelina et al. showed that naphtho- or benzo-xanthene linkers (larger P···B distance) can drop the CO_2_ hydrogenation barrier by up to 19.2 kcal compared to the parent flexible dimethylxanthene-based FLP.^17^ Similarly, Patel and Ganguly computationally studied a phosphine-borane pair in a saturated adamantane cage and demonstrated that less steric strain at reactive sites facilitates the substrate–catalyst interaction and accelerates all steps of the reduction cycle.^98^ Apart from fixing the Lewis acid and base centers, it is also beneficial to fix their substituent groups, which otherwise may tend to bind to the centers (as in the systems **2**, **8**, and **9** shown in Figure 2).

**Table 2:** Energy Components (in kcal/mol) for the Binding between FLP and CO_2_ Based on the BLW-ED Analysis

system	Δ*E*_def_	Δ*E*_F_	Δ*E*_pol_	Δ*E*_CT_	Δ*E*_int_	Δ*E*_b_	Δρ
**1**N	70.11	150.84	–94.99	–123.38	–67.53	2.58	–0.199
**2**N	95.24	133.79	–94.42	–117.75	–78.37	16.86	–0.187
**3**N	72.25	141.81	–97.61	–115.44	–71.25	1.00	–0.210
**4**N	104.74	159.99	–132.16	–135.05	–107.22	–2.48	–0.167
**5**N	86.80	131.30	–85.93	–111.83	–66.46	20.34	–0.205
**6**N	123.63	149.69	–119.78	–131.91	–101.99	21.63	–0.166
**7**N	96.49	175.98	–139.50	–132.17	–95.70	0.80	–0.199
**8**N	83.34	131.24	–88.71	–106.81	–64.29	19.05	–0.207
**9**N	116.89	154.12	–130.28	–128.05	–104.21	12.69	–0.165
**1**P	82.19	150.41	–73.97	–152.41	–75.97	6.23	–0.496
**2**P	121.85	180.88	–113.36	–172.37	–104.85	17.00	–0.620
**3**P	86.36	165.16	–92.77	–161.25	–88.86	–2.49	–0.600
**4**P	114.67	192.99	–134.13	–181.43	–122.57	–7.90	–0.576
**5**P	115.32	169.12	–99.49	–167.50	–97.86	17.45	–0.644
**6**P	146.74	201.40	–144.26	–186.27	–129.13	17.61	–0.613
**7**P	99.08	191.50	–136.21	–171.64	–116.35	–17.27	–0.613
**8**P	120.76	168.56	–108.43	–162.15	–102.02	18.75	–0.650
**9**P	151.02	209.48	–159.00	–183.67	–133.19	17.83	–0.629

Results from the BLW energy decomposition analysis of the FLP-CO_2_ adducts are compiled in Table 2. The large deformation energy costs result in very small (negative) or even positive binding energies, suggesting the overall instability of these complexes. Abstracting the deformation energy, which can be partially minimized by rational design as we discussed above, we examined the interaction energy (eq 1), which ranges from −64 kcal/mol in **8**N to −133 kcal/mol in **9**P, confirming the strong chemical interaction between FLPs and CO_2_. Notably, all energy components have large numbers. On one hand, the high and positive values of the frozen energy term indicate the strong Pauli repulsion, which overwhelms the otherwise stabilizing electrostatic and electron correlation contributions. On the other hand, the very stabilizing polarization and charge transfer interactions collectively offset the frozen energy, leading to high overall interaction energies. The strong interaction is also reflected in the large electron flow from FLPs to CO_2_ by population analyses (last column Δρ in Table 2).

Figure 5a shows the correlation between total interaction energy and the charge transfer energy. We observe two trendlines, one corresponding to N bases and the other to P bases. The two trendlines have similar slopes. For the same total interaction energy, the P bases are much more efficient than the N bases in charge transfers, in alignment with the lower electronegativity of P than that of N. Since the charge transfer interaction is an indicator of chemical reactivity, we also examined its correlation with the activation of CO_2_ in terms of C=O bond stretching, which is shown in Figure 5b. While P bases exhibit stronger charge transfer energies, they are less effective in activating CO_2_ than N bases.

**Figure 5:**
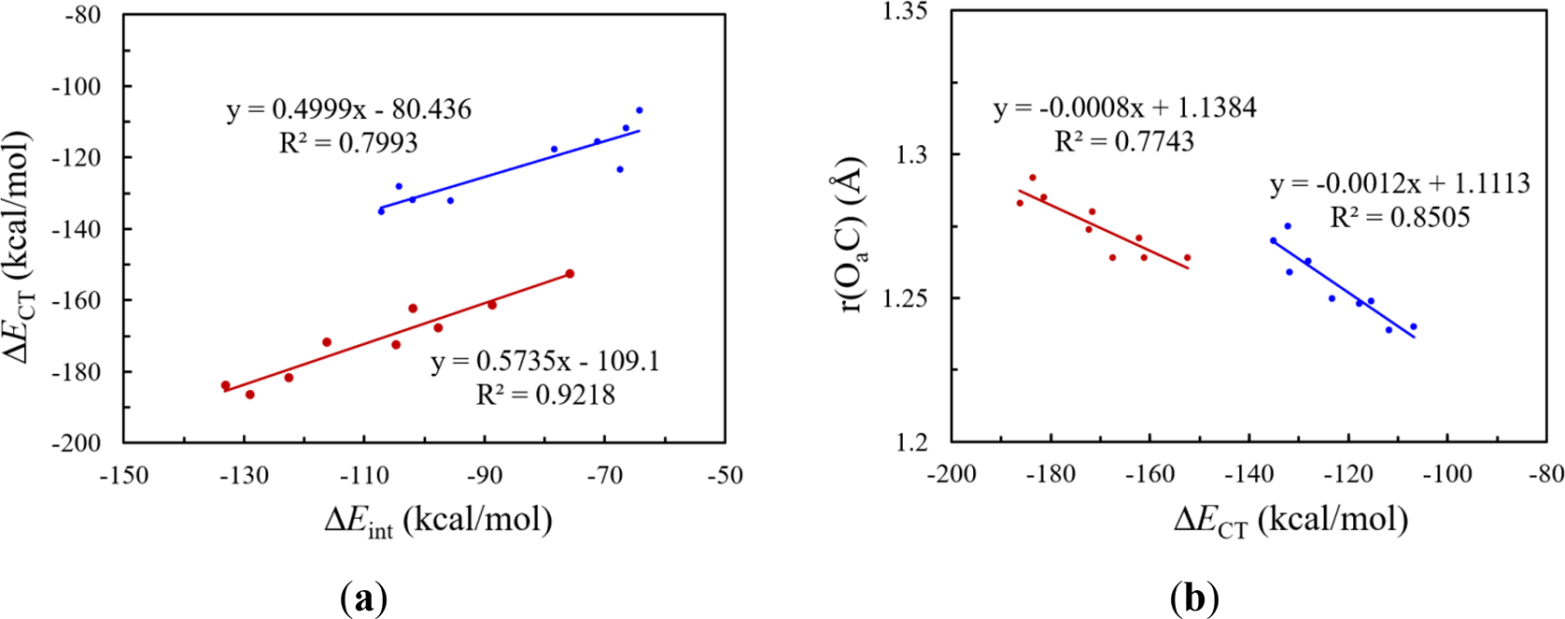
Correlations between (a) the interaction energy (Δ*E*_int_) and the charge transfer energy (Δ*E*_CT_) for the B/N FLPs (in blue) and the B/P FLPs (in brown) and (b) the charge transfer energy (Δ*E*_CT_) and the bond length of O_a_C in CO_2_.

Previous studies used frontier orbitals and charge analyses to explain the FLP reactivity. Both natural bond orbital (NBO) and EDA studies show the key charge transfer interactions including the donation from the base lone pair of N/P into the CO_2_ π* orbital and back-donation from an oxygen lone pair into the Lewis acid’s empty orbital.^34,35^ Such a mechanistic interpretation can be visually observed by the electron density difference (EDD) maps based on our present BLW computations. Figure 6 exemplifies the electron density changes due to the polarization and charge transfers, where the brown color shows the increase in electron density and the cyan color refers to the decrease in electron density. The polarization step essentially prepares for subsequent electron transfer. For instance, within CO_2_, electron density moves from the central carbon atoms to the terminal oxygen atoms (particularly the one interacting with the Lewis acid B), while the electron density around atom B moves away from the CO_2_ side. Similarly, the electron density around atom N or P polarizes toward the direction of the C atom of CO_2_. Both the polarization and the subsequent charge transfer interactions considerably stabilize the FLP complexes. Of course, the dual cooperativity in these processes is obvious, namely, the Lewis base donates electrons into CO_2_ and the Lewis acid withdraws from it. However, the former outperforms the latter, leading to the generation of the radical anion CO_2_^•–^, which is an activated species.

**Figure 6:**
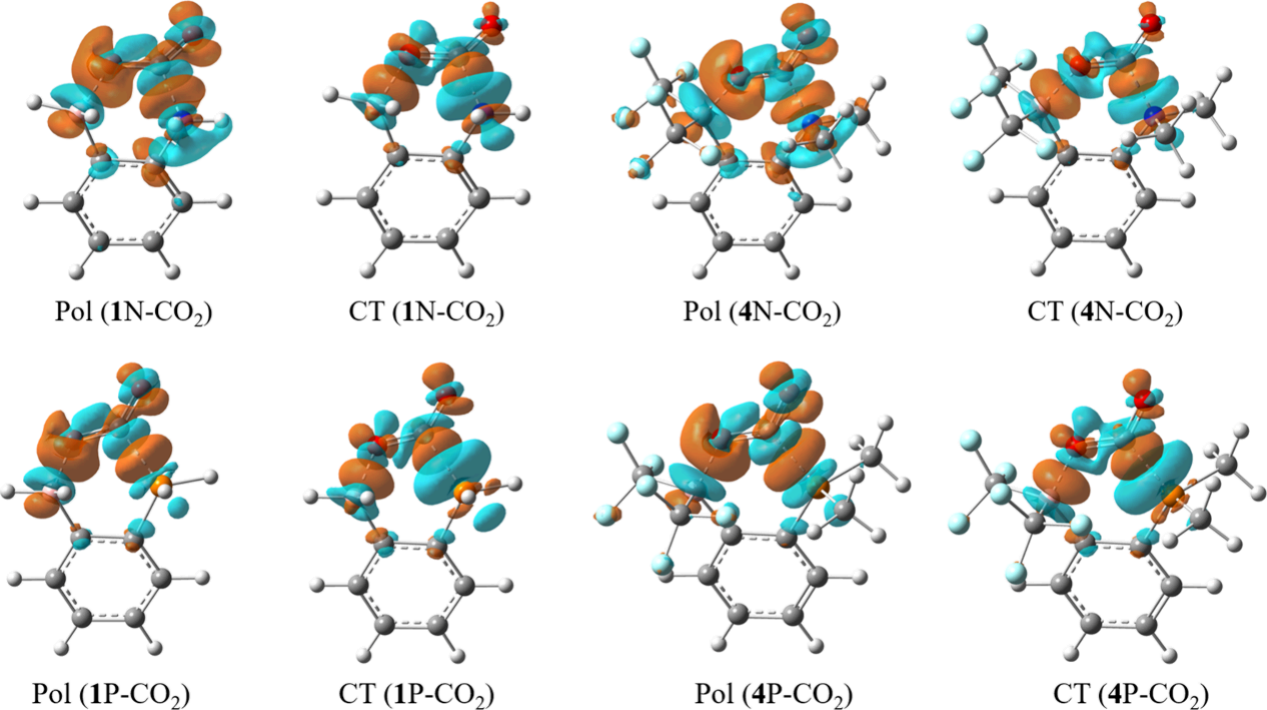
Electron density difference (EDD) maps showing the polarization and charge transfer in complexes of **1**X and **4**X (X = N/P, with an isovalue of 0.005 e Å^–3^).

### Intramolecular Electric Field (EF) and Electron Transfer (ET) in Activation Barriers

Grimme et al.^32^ suggested that there are intramolecular electric fields in FLPs, which are generated between the positively charged Lewis acids and the negatively charged Lewis bases and may play a role in the activation of small molecules such as H_2_ and CO_2_. In the present study, defining FLP and CO_2_ as two blocks to construct a BLW can effectively disable the ET interactions but retain the EF effect. Thus, performing both regular DFT and BLW computations along the reaction coordinate can allow us to trace out reaction paths on potential energy surfaces and thus provide an additional layer of information.

Here, we took the example of **1** (Figure 4) to explore the impacts of both EF and ET on the CO_2_ activation barriers, which are 10.75 and 13.26 kcal/mol for **1**N and **1**P, respectively. At the ground state, both **1**N-CO_2_ and **1**P-CO_2_ adducts are essentially van der Waals complexes and there are little charge transfer interactions. BLW computations show that the charge transfer energies are −0.82 and −1.02 kcal/mol, compared with the total binding energies of −4.39 and −3.07 kcal/mol for **1**N-CO_2_ and **1**P-CO_2_, respectively. At the transition states, however, CO_2_ is partially activated, implying considerable charge transfer interactions between FLPs and CO_2_. Indeed, BLW computations result in the charge transfer energies of −116.97 and −48.16 kcal/mol, respectively. These values are smaller in magnitude than those in the metastable adducts (−123.38 and −75.97 kcal/mol in Table 2), but significant and comparable. These results imply that both adducts would be unstable, and the energy curves along the reaction coordinates would rise monotonously. In other words, without ET, CO_2_ cannot be activated by FLPs via its EF alone. As the last evidence, we performed BLW optimizations for the **1**N-CO_2_ and **1**P-CO_2_ adducts starting from the activated structures derived at the regular DFT level. The BLW optimized geometries are shown in Figure 7. Obviously, these geometries where charge transfer interaction between **1**X and CO_2_ are completely quenched correspond to typical van der Waals complexes (as exemplified in Figure 4 for **1**N) where CO_2_ remains linear and one O binds to B and C binds to N/P, with the binding energies of 8.52 and 8.73 kcal/mol, respectively.

**Figure 7:**
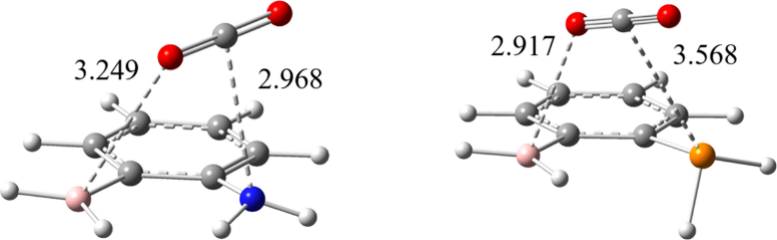
Optimal geometries of **1**X-CO_2_ (X = N and P) adducts with the charge transfer interactions between **1**X (X = N (left) and P (right)) and CO_2_ are disabled with the BLW method at the M06-2X/6-311+G­(d,p) level.

More insights into the interactions between FLPs and CO_2_ at the orbital level can be gained by the “*in situ*” orbital correlation diagrams, as exemplified with **1**N-CO_2_ in Figure 8 where the dashed red frame corresponds to the “*in situ*” orbital correlations. When **1**N and CO_2_ are put together and form a BLW state, the orbital energy levels change from the free state to the BLW state. For **1**N, the HOMO energy lowers but the LUMO energy increases. For CO_2_, the HOMO energy changes little but the LUMO energy is up. These orbital energy changes can be regarded as the field effects (including both electrostatic and Pauli repulsion, in addition to the electron correlations), leading to the increased HOMO–LUMO gaps. The mutual HOMO–LUMO interactions (charge transfer interactions) result in the decreasing of the occupied orbital energies, though in the figure the reductions seem insignificant. In detail, the HOMO of **1**N interacts with the LUMO of CO_2_ (corresponding to the electron transfer from N of **1**N to the C of CO_2_), leading to the energy reduction of the occupied orbital by 0.45 eV. In contrast, the HOMO of CO_2_ interacts with the LUMO of **1**N (corresponding to the electron transfer from the O of CO_2_ to B of **1**N), leading to the energy reduction of the occupied orbital by 0.21 eV. The electron transfer from **1**N to CO_2_ thus is superior to the transfer from CO_2_ to **1**N.

**Figure 8:**
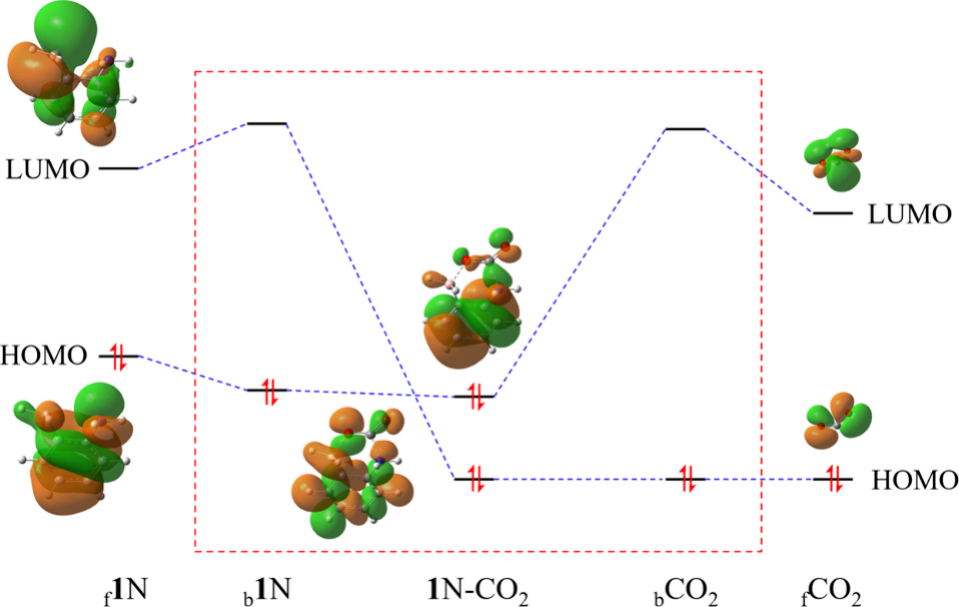
“*In situ*” orbital correlation diagram for the **1**N-CO_2_ adduct, where the subscripts f and b refer to the free state and BLW state, respectively.

## Conclusions

CO_2_ mitigation is one of the grand challenges for the 21st century, and the integrated CO_2_ capture and conversion is a plausible way. Due to the high stability of CO_2_, catalysts are required. Thus, it is fundamental to understand how catalysts play the critical role and what the governing factors are in chemical transformations such as the reduction of CO_2_. The knowledge garnered from basic research would allow us to understand the mechanisms and control transformations by rationally designed catalysts with superior performance and economic benefits. Here, we studied the absorption of CO_2_ by metal-free frustrated Lewis pairs (FLPs) with B as the Lewis acid center and N/P as the Lewis base center. Our energy decomposition analysis based on the BLW method showed that the very low overall binding energy results from the very strong and stabilizing chemical interactions (including both polarization and charge transfer interactions as discussed in the literature) offset by the equally very strong but destabilizing deformation energy and Pauli repulsion. While the Pauli repulsion is inevitable, the deformation cost can be minimized by a rigid design of FLPs. For instance, both the Lewis acid and base can be fixed at solid surfaces. While the substituent groups bonding to the acid and base centers (such as B and N/P in this study) can tune the FLP’s HOMO and LUMO energies, i.e., more electron-withdrawing groups on B or electron-donating groups on N/P, ideally, these substituent groups also should be fixed as otherwise they may interfere with the interaction between the acid and base centers.

## Supplementary Material



## Notes

Optimal geometries with energies at the M06-2X/6-311+G­(d,p) theoretical level (PDF)
