# Supplementary material for: Energy Decomposition Analysis of the Activation of CO2 by Frustrated Lewis Pairs
Source: J Phys Chem A. 2025 Oct 23;129(44):10202–12. doi: 10.1021/acs.jpca.5c06202 (PMC12598860; doi:10.1021/acs.jpca.5c06202)
Supplement: Supplementary file 1 [file jp5c06202_si_001.pdf]

Supplemental Information

Energy Decomposition Analysis of the Activation of CO_2 by Frustrated Lewis Pairs (FLPs)

Manoj Wijesingha, Xinru Peng, Nathan Hoang, Nicholas Jamieson, Sherine O. Obare*, and Yirong Mo*

Department of Nanoscience, Joint School of Nanoscience & Nanoengineering, University of North Carolina at Greensboro, Greensboro, NC 27401 (USA)

E-mails: soobare@uncg.edu (SO) and y_mo3@uncg.edu (YM)

Optimal geometries:

CO_2
E = -188.5748797 a.u.

C	6.0	0.000000	0.000000	0.000000
O	8.0	0.000000	0.000000	1.154863
O	8.0	0.000000	0.000000	-1.154863

1N
E = -312.987791662 a.u.

C	-2.559891	0.239075	-0.721313
C	-2.292972	-1.105362	-0.440998
C	-1.171501	-1.477209	0.274960
C	-0.269814	-0.505891	0.744521
C	-0.518345	0.869825	0.482976
C	-1.675103	1.193880	-0.260262
H	-3.440029	0.518948	-1.285528
H	-2.972802	-1.874407	-0.791215
H	-0.974953	-2.525470	0.473563
H	-1.863767	2.242739	-0.464031
N	0.860041	-0.917273	1.410506
H	0.859936	-1.841639	1.808650
H	1.386437	-0.225040	1.917298
B	0.377623	2.002006	0.977478
H	1.343460	1.815802	1.655006
H	0.106341	3.125707	0.691401

1N'
E = -312.9801586 a.u.

C	-1.961541	0.607687	0.000065
C	-1.855608	-0.786139	-0.000069
C	-0.605344	-1.406843	-0.000249
C	0.474642	-0.545123	-0.000322

C	0.422740	0.837516	-0.000160
C	-0.834917	1.433251	0.000059
H	-2.949842	1.053846	0.000212
H	-2.754262	-1.391016	0.000007
H	-0.503989	-2.486249	-0.000250
H	-0.955887	2.510701	0.000156
N	1.922865	-0.778669	0.000068
B	2.033498	0.963405	0.000380
H	2.571694	1.299764	1.018569
H	2.572861	1.300141	-1.017062
H	2.276243	-1.247574	-0.829367
H	2.275809	-1.248056	0.829416

1N-CO_2

Ground State (Global minimum) E = -501.570505768 a.u.

C	-2.562795	0.236375	-0.716985
C	-2.304136	-1.106029	-0.426671
C	-1.178084	-1.477287	0.285024
C	-0.273691	-0.505710	0.738983
C	-0.517140	0.867550	0.474893
C	-1.670116	1.192942	-0.270323
H	-3.442683	0.516610	-1.281618
H	-2.988641	-1.874056	-0.769246
H	-0.978333	-2.525204	0.481767
H	-1.853862	2.240562	-0.484757
N	0.895508	-0.912523	1.359352
H	0.888066	-1.833164	1.770328
H	1.376453	-0.217059	1.910170
B	0.379218	1.999595	0.990993
H	1.258576	1.817667	1.776019
H	0.166322	3.115423	0.635081
C	2.027791	-0.082838	-1.239896
O	2.451954	0.857804	-0.715715
O	1.618305	-1.012824	-1.785525

2nd Minimum (Activated state) E = -501.5615811 a.u.

C	6.0	-2.832135	0.328832	-0.360475
C	6.0	-2.578910	-1.033684	-0.211429
C	6.0	-1.305507	-1.465240	0.144392
C	6.0	-0.341244	-0.490352	0.337870
C	6.0	-0.529822	0.876598	0.189183
C	6.0	-1.821788	1.268003	-0.169774
H	1.0	-3.826578	0.656588	-0.640087
H	1.0	-3.366591	-1.758471	-0.374305
H	1.0	-1.084047	-2.520478	0.265112
H	1.0	-2.031854	2.322573	-0.309501
N	7.0	1.034027	-0.849597	0.728740
H	1.0	1.205496	-1.854625	0.740432
H	1.0	1.221106	-0.449664	1.657360
B	5.0	0.770319	1.790212	0.432676
H	1.0	1.099830	1.754976	1.605712

H	1.0	0.721881	2.892827	-0.021697
C	6.0	2.120408	-0.206388	-0.246254
O	8.0	1.941787	1.027091	-0.346411
O	8.0	2.896333	-0.991369	-0.689671

Transition State between the Global State and 2nd Minimum E = -501.5533600 a.u.

C	-2.945144	0.388099	-0.000092
C	-2.730526	-0.993152	0.000046
C	-1.432756	-1.473609	0.000140
C	-0.385958	-0.553066	0.000110
C	-0.539564	0.820103	0.000066
C	-1.873941	1.265449	-0.000088
H	-3.958598	0.772087	-0.000227
H	-3.565335	-1.682474	0.000090
H	-1.236645	-2.541804	0.000264
H	-2.057254	2.334952	-0.000226
N	0.989372	-1.102204	-0.000125
H	1.144686	-1.702408	0.813181
H	1.144484	-1.701705	-0.813992
B	0.670346	1.867324	-0.000023
H	0.690419	2.528092	1.004315
H	0.690523	2.527688	-1.004637
C	2.289857	-0.149351	0.000035
O	2.040760	1.062710	0.000239
O	3.281563	-0.810518	-0.000124

2N
E = -423.6474323

C	-2.632851	0.153318	0.126698
C	-2.172556	-1.154036	-0.010140
C	-0.811708	-1.411336	-0.160687
C	0.046171	-0.321785	-0.168709
C	-0.370803	1.002159	-0.036806
C	-1.737033	1.222364	0.114260
H	-3.693881	0.338132	0.248017
H	-2.873459	-1.980209	0.003495
H	-0.429072	-2.418072	-0.269236
H	-2.109115	2.235214	0.226149
N	1.469285	-0.443101	-0.361825
B	0.892922	1.984800	-0.136328
N	2.018739	0.802626	0.146272
H	2.177800	0.722966	1.156194
H	2.906666	0.975892	-0.317812
N	2.017241	-1.588023	0.203907
H	2.938839	-1.770142	-0.170169
H	2.015488	-1.574847	1.225024
H	1.025024	2.815363	0.724962
H	1.112931	2.387076	-1.251162

2N-CO_2
E = -612.1993915 a.u.

C	3.159280	0.199894	0.262070
C	2.734328	-1.126212	0.295670
C	1.387957	-1.427705	0.132489
C	0.507885	-0.367425	-0.056274
C	0.878412	0.972098	-0.103535
C	2.243043	1.226890	0.070293
H	4.210206	0.430017	0.393120
H	3.446708	-1.927588	0.447270
H	1.043062	-2.452278	0.136321
H	2.581903	2.257107	0.060487
N	-0.939761	-0.626618	-0.300072
B	-0.210724	2.125812	-0.350397
H	-0.448782	2.289121	-1.529644
H	0.055761	3.152973	0.202867
N	-1.289626	-1.972706	0.002307
H	-1.182424	-2.071029	1.011313
H	-2.290879	-2.022829	-0.180130
N	-1.332596	-0.434810	-1.675923
H	-1.112436	0.539143	-1.898230
H	-0.700077	-1.024182	-2.216780
C	-1.862242	0.424834	0.547684
O	-1.555175	1.611502	0.311601
O	-2.683264	-0.108357	1.219751

3N

E = -470.2105501 a.u.

C	2.874025	0.815516	0.000640
C	2.864433	-0.581601	-0.000218
C	1.659303	-1.286367	-0.000493
C	0.516616	-0.506352	0.000186
C	0.476091	0.876050	0.001017
C	1.690131	1.557336	0.001247
H	3.828357	1.330478	0.000772
H	3.803070	-1.122819	-0.000735
H	1.637994	-2.370905	-0.001203
H	1.735357	2.641082	0.001875
N	-0.899106	-0.877320	0.000018
B	-1.144953	0.932675	-0.000026
C	-1.315949	-1.605318	-1.208916
H	-0.907593	-2.620727	-1.202411
H	-2.405798	-1.652094	-1.237634
H	-0.955775	-1.076655	-2.088080
C	-1.316002	-1.604493	1.209457
H	-2.405847	-1.651318	1.238159
H	-0.907478	-2.619839	1.203714
H	-0.955886	-1.075186	2.088260
C	-1.885225	1.379140	-1.350957
H	-2.911400	0.998042	-1.411938
H	-1.963609	2.472126	-1.347564
H	-1.365090	1.111006	-2.274584
C	-1.888971	1.378672	1.348887

H	-1.968058	2.471598	1.345449
H	-2.915075	0.996902	1.406849
H	-1.371378	1.110686	2.273975

3N-CO_2
E = -658.7875046 a.u.

C	-3.192021	-0.318349	-0.200210
C	-2.740716	-1.635060	-0.210169
C	-1.382830	-1.896623	-0.099310
C	-0.496561	-0.825416	0.017336
C	-0.899660	0.506630	0.014415
C	-2.281661	0.722318	-0.098567
H	-4.252091	-0.108680	-0.282518
H	-3.437844	-2.458349	-0.304744
H	-1.048722	-2.924335	-0.111369
H	-2.641633	1.745695	-0.113131
N	0.959874	-1.098409	0.235477
B	0.104158	1.773342	0.063823
C	1.382244	-2.454408	-0.202140
H	0.911429	-3.195620	0.439463
H	1.096583	-2.599375	-1.240998
H	2.462418	-2.515847	-0.114133
C	1.272723	-0.941345	1.692581
H	0.686942	-1.674919	2.245681
H	2.340793	-1.109718	1.836118
H	1.002740	0.065038	1.996382
C	0.149052	2.544358	1.480561
H	0.952158	3.288432	1.484439
H	-0.789670	3.091449	1.621379
H	0.273959	1.920624	2.372221
C	-0.133395	2.769062	-1.182128
H	-0.111958	2.253436	-2.147863
H	-1.099745	3.279180	-1.106689
H	0.637308	3.545878	-1.202843
C	1.874696	-0.008981	-0.533591
O	1.575486	1.160279	-0.213727
O	2.732788	-0.479511	-1.212712

4N
E = -1065.7119367 a.u.

C	-3.264414	0.011717	-1.584685
C	-3.765553	0.001487	-0.278692
C	-2.911732	-0.007165	0.825094
C	-1.566599	-0.004932	0.508734
C	-1.028844	0.005096	-0.761600
C	-1.893973	0.013921	-1.848480
H	-3.966162	0.018291	-2.410541
H	-4.837461	0.000380	-0.122131
H	-3.291569	-0.014953	1.840108
H	-1.532431	0.021974	-2.869666
N	-0.349841	-0.011409	1.343655

B	0.452062	0.001236	-0.157651
C	-0.228176	1.191278	2.209020
H	-0.976629	1.129248	3.001387
H	0.771558	1.213811	2.640762
H	-0.395163	2.085325	1.615552
C	-0.227643	-1.228310	2.189048
H	0.772663	-1.258480	2.618997
H	-0.975116	-1.178786	2.983209
H	-0.395939	-2.112518	1.581432
C	1.344620	1.342180	-0.348482
C	1.347157	-1.335088	-0.368683
F	0.611711	2.485142	-0.324998
F	2.282746	1.493781	0.620965
F	1.999721	1.358892	-1.520311
F	2.004755	-1.333153	-1.539090
F	2.283567	-1.499981	0.600408
F	0.615919	-2.479315	-0.364096

4N-CO_2
E = -1254.2955594 a.u.

C	-1.144852	3.199338	-0.198109
C	-2.464528	2.761449	-0.193439
C	-2.744957	1.405792	-0.098185
C	-1.685888	0.504150	-0.016987
C	-0.354553	0.902439	-0.025154
C	-0.110354	2.279557	-0.118672
H	-0.924411	4.257540	-0.267514
H	-3.280972	3.469490	-0.262340
H	-3.777507	1.088099	-0.092457
H	0.916481	2.624038	-0.130505
N	-1.989065	-0.953241	0.181833
B	0.847112	-0.151641	0.017095
C	-3.292034	-1.359822	-0.422571
H	-4.097353	-0.881400	0.126772
H	-3.302275	-1.063710	-1.468898
H	-3.378835	-2.438386	-0.343605
C	-2.023498	-1.244999	1.660060
H	-2.818449	-0.642322	2.096680
H	-2.224549	-2.308552	1.792529
H	-1.064002	-0.974093	2.092987
C	1.717058	-0.034589	1.379813
C	1.767409	-0.024916	-1.318165
F	2.688898	-0.952247	1.502685
F	0.931505	-0.186300	2.490648
F	2.303760	1.173699	1.513797
F	2.763272	-0.924502	-1.387239
F	1.009729	-0.220533	-2.430767
F	2.340976	1.191201	-1.466888
C	-0.862989	-1.902247	-0.406378
O	0.305166	-1.570195	-0.036388
O	-1.243714	-2.848380	-1.008033

5N
E = -502.2796206 a.u.

C	-2.386072	-1.586307	-0.101578
C	-2.898631	-0.300217	0.049413
C	-2.043359	0.793153	0.169175
C	-0.679354	0.543712	0.132537
C	-0.123663	-0.725511	-0.016827
C	-1.007029	-1.794789	-0.134537
H	-3.064207	-2.426320	-0.197321
H	-3.970558	-0.143272	0.071510
H	-2.419039	1.801492	0.286703
H	-0.619737	-2.801589	-0.257241
N	0.312830	1.575750	0.292227
B	1.485649	-0.658235	0.027042
N	1.536043	1.009026	-0.238743
H	1.593596	1.179151	-1.249103
H	2.327552	1.454849	0.220827
N	-0.050875	2.798595	-0.261544
H	0.528998	3.543003	0.102396
H	-0.056985	2.790127	-1.282736
C	2.278053	-1.368401	-1.182765
H	3.350492	-1.138400	-1.184958
H	2.201186	-2.455962	-1.078910
H	1.876497	-1.124719	-2.173881
C	2.104793	-0.909411	1.496653
H	1.973581	-1.960298	1.773671
H	3.182502	-0.707580	1.541980
H	1.613465	-0.316275	2.275859

5N-CO_2
E = -690.8260048 a.u.

C	-3.221213	-0.195101	-0.158816
C	-2.820511	-1.529070	-0.200438
C	-1.473080	-1.845082	-0.121098
C	-0.560426	-0.802913	0.002356
C	-0.900596	0.543763	0.024855
C	-2.275028	0.814025	-0.061123
H	-4.274250	0.053972	-0.219569
H	-3.551828	-2.321513	-0.299270
H	-1.143023	-2.871974	-0.175276
H	-2.599124	1.849046	-0.063689
B	0.173799	1.749730	0.049805
N	0.881717	-1.134856	0.210735
C	0.456479	2.413389	1.496159
H	0.715230	1.744014	2.325859
H	1.263399	3.149278	1.423162
H	-0.440144	2.954743	1.816588
C	-0.124579	2.851414	-1.083201
H	-0.284592	2.410510	-2.071927
H	-1.015987	3.438643	-0.838703

H	0.708154	3.556995	-1.161808
N	1.162855	-0.996697	1.630324
H	0.722802	-0.126438	1.927383
H	2.173198	-0.879498	1.721117
N	1.182382	-2.461359	-0.197107
H	2.148270	-2.446805	-0.521592
H	1.101118	-3.038517	0.635636
C	1.894372	-0.117943	-0.584767
O	1.566962	1.069107	-0.446532
O	2.828864	-0.677308	-1.070738

6N

E = -1097.7862332 a.u.

C	-2.646939	-2.137673	0.460160
C	-3.570381	-1.101641	0.335198
C	-3.151616	0.205555	0.103193
C	-1.786667	0.426618	0.003298
C	-0.837827	-0.583859	0.118380
C	-1.282027	-1.881228	0.352107
H	-2.994469	-3.148032	0.637795
H	-4.630050	-1.311820	0.417147
H	-3.853528	1.023150	0.003933
H	-0.565387	-2.689705	0.441496
N	-1.215805	1.735749	-0.178574
B	0.612768	0.044069	-0.032878
N	0.167451	1.496823	-0.606780
H	0.213056	1.475408	-1.633004
H	0.751185	2.261941	-0.264818
N	-1.938202	2.557690	-1.024076
H	-1.689827	3.529464	-0.899502
H	-1.924317	2.266665	-2.001554
C	1.549753	-0.648565	-1.155025
C	1.406817	0.316219	1.352716
F	2.310168	1.333919	1.221794
F	0.596259	0.696452	2.362013
F	2.094809	-0.751454	1.786865
F	2.796472	-0.134083	-1.240430
F	1.685537	-1.971210	-0.982903
F	1.011057	-0.477156	-2.401924

6N-CO_2

E = -1286.332158 a.u.

C	-1.178795	3.212040	0.057963
C	-2.495171	2.762528	-0.010534
C	-2.761006	1.403289	-0.058628
C	-1.685580	0.523773	-0.039589
C	-0.358358	0.922569	0.012475
C	-0.130025	2.305438	0.062466
H	-0.972192	4.274558	0.099323
H	-3.316685	3.467595	-0.028801
H	-3.778411	1.047868	-0.127787

H	0.891188	2.664220	0.100339
B	0.841939	-0.128305	-0.040585
N	-1.979846	-0.943651	0.032811
C	1.583333	-0.272996	1.396038
C	1.889296	0.207918	-1.235884
F	0.687272	-0.648334	2.375930
F	2.112603	0.886428	1.823678
F	2.556145	-1.192075	1.443992
F	2.843458	-0.726405	-1.393644
F	1.252350	0.298509	-2.428733
F	2.533138	1.384991	-1.061607
N	-2.046304	-1.308371	1.439228
H	-1.227018	-0.907105	1.899430
H	-1.968229	-2.325543	1.485756
N	-3.226657	-1.244615	-0.588093
H	-3.154910	-2.203955	-0.924345
H	-3.922545	-1.195873	0.151730
C	-0.864706	-1.875682	-0.651954
O	0.314132	-1.515176	-0.397594
O	-1.295367	-2.842955	-1.186282

7N

E = -2081.7906158 a.u.

C	-0.057819	-2.807198	3.101267
C	-1.092591	-3.546421	2.539709
C	-1.659263	-3.151534	1.334440
C	-1.233475	-1.996003	0.666090
C	-0.149438	-1.278805	1.207251
C	0.424213	-1.704670	2.415127
H	0.387603	-3.106012	4.042289
H	-1.451769	-4.443244	3.031220
H	-2.433319	-3.765669	0.899274
H	1.267640	-1.155793	2.824901
N	-1.824498	-1.573051	-0.587619
B	0.485696	-0.015303	0.547601
C	-3.280080	-1.199420	-0.452511
C	-1.336241	-2.353395	-1.789202
C	2.031401	0.042786	0.230888
C	2.706518	1.248345	-0.013397
C	2.844600	-1.101113	0.186292
C	4.062088	1.324285	-0.283280
C	4.201165	-1.058164	-0.090142
C	4.813411	0.161601	-0.325160
C	-0.375034	1.294233	0.370923
C	-0.982278	1.889401	1.465709
C	-0.563673	1.914259	-0.854126
C	-1.756501	3.033957	1.358316
C	-1.355825	3.036731	-1.009682
C	-1.953446	3.600814	0.109846
F	-0.832187	1.357933	2.682491
F	-2.323533	3.577171	2.429033

F	-2.705184	4.684110	-0.017450
F	-1.533146	3.591608	-2.203003
F	0.047995	1.425605	-1.939096
F	2.068328	2.415937	0.020560
F	4.647270	2.494663	-0.498577
F	6.106431	0.216014	-0.583685
F	4.919610	-2.172608	-0.129294
F	2.347822	-2.314630	0.394067
C	-2.129051	-3.638970	-2.080387
H	-1.669465	-4.154951	-2.927618
H	-2.114689	-4.323576	-1.229364
H	-3.164295	-3.430252	-2.351078
C	-1.310580	-1.486418	-3.065210
H	-2.276545	-1.394669	-3.553665
H	-0.930221	-0.490217	-2.839803
H	-0.635081	-1.951202	-3.787354
C	0.128185	-2.755595	-1.559089
H	0.273175	-3.387916	-0.684173
H	0.477686	-3.302642	-2.436110
H	0.753832	-1.865250	-1.455389
C	-4.247478	-2.306653	0.035751
H	-4.139362	-2.489693	1.105159
H	-5.271248	-1.959788	-0.126739
H	-4.131367	-3.249334	-0.496488
C	-3.841386	-0.639705	-1.765593
H	-4.025711	-1.411313	-2.513020
H	-4.801776	-0.168662	-1.544778
H	-3.181223	0.121216	-2.183855
C	-3.386380	-0.051742	0.562680
H	-2.980849	0.863495	0.132985
H	-4.439235	0.123189	0.796930
H	-2.867683	-0.275704	1.497529

7N-CO_2
E = -2270.3692219 a.u.

C	0.718239	-1.667439	3.490876
C	2.032925	-2.051077	3.246177
C	2.623852	-1.746660	2.032467
C	1.915025	-1.055985	1.043937
C	0.574559	-0.719172	1.242972
C	0.012317	-1.025583	2.491819
H	0.247926	-1.888102	4.441449
H	2.603917	-2.586209	3.995057
H	3.642110	-2.054617	1.882259
H	-1.021066	-0.751133	2.670053
N	2.631672	-0.708593	-0.252490
B	-0.356106	-0.178380	0.044464
C	3.108116	-2.071291	-1.009235
C	3.742642	0.428715	0.044044
C	-0.569523	1.442725	-0.049614
C	-1.057884	1.995530	-1.229738

C	-0.303389	2.353728	0.962493
C	-1.241015	3.357279	-1.414878
C	-0.473231	3.722441	0.820599
C	-0.942374	4.227688	-0.379916
C	-1.795853	-0.940447	-0.012647
C	-2.862777	-0.480822	0.752144
C	-2.077310	-2.058358	-0.788933
C	-4.122914	-1.056510	0.746268
C	-3.325345	-2.663958	-0.827868
C	-4.357368	-2.159127	-0.057071
F	-2.696421	0.570800	1.571991
F	-5.100533	-0.568115	1.504245
F	-5.554624	-2.731740	-0.079130
F	-3.536124	-3.731823	-1.593247
F	-1.134611	-2.633266	-1.548562
F	-1.388716	1.202842	-2.252728
F	-1.704760	3.835710	-2.565772
F	-1.109281	5.535508	-0.535359
F	-0.191985	4.552753	1.822971
F	0.140375	1.936960	2.158211
C	4.649011	0.735861	-1.159886
H	5.272361	1.577903	-0.852046
H	5.326100	-0.081304	-1.400912
H	4.106482	1.037943	-2.046059
C	2.979307	1.693840	0.461724
H	2.433947	2.156033	-0.357728
H	2.298431	1.487439	1.289177
H	3.716771	2.414224	0.820532
C	4.671857	0.087482	1.221292
H	5.209767	-0.850142	1.110834
H	5.420777	0.880847	1.240123
H	4.158341	0.109488	2.179261
C	4.479812	-2.585507	-0.559378
H	4.518112	-2.876757	0.485872
H	4.653001	-3.497065	-1.134894
H	5.299336	-1.908188	-0.781929
C	3.164881	-1.846183	-2.530048
H	3.785705	-1.003280	-2.821764
H	3.593691	-2.751219	-2.962175
H	2.172157	-1.721380	-2.964256
C	2.068680	-3.166489	-0.739460
H	1.055441	-2.833414	-0.954327
H	2.299873	-3.991591	-1.416552
H	2.114453	-3.544481	0.280361
C	1.606040	-0.088214	-1.301285
O	0.435075	-0.583998	-1.213292
O	2.018878	0.687197	-2.096951

8N

E = -659.4484212 a.u.

C	-3.302033	1.176934	0.157148

C	-2.425455	2.244517	0.298688
C	-1.045052	2.055275	0.192204
C	-0.584337	0.776310	-0.088614
C	-1.436331	-0.325704	-0.186310
C	-2.801756	-0.109110	-0.065776
H	-4.369882	1.338982	0.246974
H	-2.807766	3.236252	0.510478
H	-0.366484	2.880679	0.357843
H	-3.487123	-0.949197	-0.126824
N	0.795810	0.392250	-0.276118
B	-0.575465	-1.665327	-0.341843
C	-0.278309	-2.055111	-1.879125
H	0.151848	-1.234369	-2.461874
H	0.357017	-2.939629	-2.003323
H	-1.239999	-2.297906	-2.344102
C	-0.997822	-2.915980	0.579593
H	-1.309387	-2.660613	1.596649
H	-1.861065	-3.393069	0.101775
H	-0.229585	-3.697237	0.644847
N	1.709739	1.453856	0.151008
N	0.893805	-0.921604	0.323673
C	1.801484	2.337302	-1.024137
H	2.321297	3.247712	-0.721602
H	2.349445	1.852171	-1.842635
H	0.812051	2.601501	-1.387008
C	3.073385	1.018860	0.434711
H	3.644977	1.928350	0.622499
H	3.138484	0.409523	1.331708
H	3.540863	0.491579	-0.408445
C	0.804017	-0.903790	1.806915
H	0.889564	-1.931443	2.155834
H	1.591588	-0.293791	2.243727
H	-0.167263	-0.504288	2.092268
C	2.053368	-1.711446	-0.142527
H	2.232480	-1.472352	-1.187661
H	2.943031	-1.523008	0.454396
H	1.780810	-2.763068	-0.052884

8N-CO_2
E = -847.9970285 a.u.

C	2.755490	2.320545	0.141811
C	1.536996	2.995682	0.175336
C	0.352587	2.279507	0.094737
C	0.417709	0.892181	-0.025480
C	1.605126	0.170830	-0.054604
C	2.777824	0.939307	0.024725
H	3.684110	2.875987	0.205609
H	1.504936	4.074171	0.269635
H	-0.601638	2.784544	0.143215
H	3.731735	0.424093	-0.007558
N	-0.865843	0.090455	-0.014863

B	1.694526	-1.436635	-0.165434
C	1.815983	-2.185797	1.256770
H	1.086472	-1.851786	2.000117
H	1.710334	-3.269469	1.134141
H	2.812055	-2.010959	1.678486
C	2.783471	-1.930534	-1.245463
H	2.682841	-1.433346	-2.215850
H	3.804278	-1.752768	-0.890177
H	2.692244	-3.008315	-1.413220
N	-0.973154	-0.494125	1.284289
N	-1.978962	0.971090	-0.352330
C	-1.025361	0.444504	2.392158
H	-1.955445	1.025478	2.431330
H	-0.942667	-0.153983	3.300181
H	-0.175777	1.123772	2.359795
C	-1.793408	-1.691686	1.459104
H	-2.778308	-1.473141	1.883875
H	-1.937929	-2.228180	0.524322
H	-1.251295	-2.354084	2.137859
C	-3.296567	0.426787	-0.039138
H	-4.009347	1.173853	-0.390291
H	-3.510082	-0.521829	-0.539680
H	-3.431625	0.333179	1.035860
C	-1.917184	1.476321	-1.735142
H	-0.888692	1.513498	-2.087700
H	-2.497295	0.858508	-2.421541
H	-2.307484	2.496758	-1.725230
C	-0.725445	-1.182387	-1.065879
O	0.282404	-1.869422	-0.841155
O	-1.643861	-1.273865	-1.818756

9N

E = -1254.9516712 a.u.

C	-0.704838	3.611447	0.075839
C	-2.027482	3.204425	-0.050465
C	-2.367691	1.850374	-0.078812
C	-1.340525	0.926234	0.049470
C	-0.002536	1.310414	0.120791
C	0.313955	2.659561	0.141643
H	-0.464079	4.667306	0.095004
H	-2.813037	3.945192	-0.142184
H	-3.394132	1.544420	-0.224444
H	1.351475	2.971637	0.192332
N	-1.474218	-0.508768	0.106530
B	0.874017	-0.004935	0.040601
C	1.357081	-0.526387	1.510767
C	2.172258	0.086461	-0.939624
N	-2.809513	-0.943373	-0.304884
N	-0.295196	-1.013059	-0.600083
C	-3.607324	-0.812051	0.931313
H	-4.657668	-0.932247	0.662995

H	-3.320256	-1.571710	1.668594
H	-3.468414	0.166416	1.381594
C	-2.925633	-2.346018	-0.699884
H	-3.992011	-2.523591	-0.840339
H	-2.442529	-2.557772	-1.649509
H	-2.564118	-3.041433	0.068813
C	-0.386434	-0.804037	-2.081801
H	0.507130	-1.226562	-2.536793
H	-1.274290	-1.292975	-2.469746
H	-0.435481	0.263420	-2.281664
C	0.018452	-2.437880	-0.281432
H	-0.218664	-2.610156	0.765406
H	-0.541485	-3.109860	-0.923847
H	1.083110	-2.582199	-0.451218
F	0.346773	-0.902816	2.330977
F	2.205371	-1.589459	1.476589
F	2.015948	0.450345	2.160062
F	3.213283	0.659612	-0.311485
F	2.619389	-1.122517	-1.386274
F	1.969365	0.819939	-2.060511

9N-CO_2
E = -1443.5117448 a.u.

C	-0.265069	3.475232	0.012692
C	1.110357	3.388858	-0.194420
C	1.728218	2.150338	-0.225425
C	0.953329	1.002621	-0.041137
C	-0.426349	1.045829	0.099451
C	-1.017127	2.320025	0.138683
H	-0.749963	4.443475	0.044425
H	1.702381	4.284056	-0.339919
H	2.786106	2.070785	-0.423965
H	-2.092895	2.387758	0.244725
N	1.677507	-0.312850	0.033200
B	-1.329017	-0.262192	0.009174
C	-2.149429	-0.247959	-1.397990
C	-2.310966	-0.464753	1.285129
N	2.672089	-0.324004	-1.040014
N	2.375457	-0.497272	1.313295
C	1.989771	-0.337871	-2.337451
H	2.766429	-0.231947	-3.094255
H	1.453829	-1.279826	-2.515269
H	1.297070	0.496549	-2.426443
C	3.687362	-1.389528	-0.972211
H	4.557450	-0.994215	-1.500440
H	3.952064	-1.605276	0.057677
H	3.355534	-2.314405	-1.446818
C	3.394020	0.515006	1.592851
H	2.974464	1.479632	1.896938
H	3.983321	0.109555	2.416268
H	4.048525	0.646784	0.734165

C	1.413912	-0.589652	2.412959
H	0.736436	-1.431900	2.276651
H	2.001228	-0.779972	3.310427
H	0.829528	0.327138	2.549643
F	-1.282874	-0.156582	-2.449036
F	-2.880393	-1.352161	-1.625959
F	-2.990321	0.801345	-1.521716
F	-3.188745	0.553804	1.431782
F	-1.609963	-0.517417	2.453249
F	-3.038641	-1.593733	1.249499
C	0.777303	-1.660342	-0.070354
O	-0.475831	-1.503834	-0.074372
O	1.422661	-2.648976	-0.129637

1P
E = -599.556963 a.u.

C	-2.390467	-0.098983	0.005712
C	-1.761389	-1.338437	-0.007455
C	-0.371307	-1.426178	-0.002267
C	0.416761	-0.275884	0.009029
C	-0.205788	0.997194	0.004874
C	-1.610994	1.049624	0.015903
H	-3.471616	-0.032605	0.008737
H	-2.352651	-2.247359	-0.014091
H	0.094190	-2.405503	0.000937
H	-2.090160	2.022978	0.027312
P	2.257436	-0.432446	-0.101421
H	2.571564	0.133159	1.157269
H	2.310308	-1.764914	0.374035
B	0.575577	2.326240	-0.022888
H	1.763977	2.352558	-0.085175
H	-0.025933	3.353169	0.011962

1P'
E = -599.5494413 a.u.

C	-2.345420	0.441936	-0.000066
C	-2.116308	-0.939190	-0.000283
C	-0.818834	-1.434898	-0.000295
C	0.215278	-0.503748	-0.000080
C	0.020037	0.884791	0.000150
C	-1.296229	1.355157	0.000148
H	-3.368052	0.803039	-0.000064
H	-2.957199	-1.622388	-0.000441
H	-0.628520	-2.502032	-0.000460
H	-1.505720	2.419148	0.000317
P	2.016537	-0.489962	0.000037
B	1.490764	1.563546	0.000374
H	1.887107	2.050591	1.020041
H	1.887265	2.050962	-1.019056
H	2.516103	-1.245728	-1.078621
H	2.515991	-1.246194	1.078421

1P-CO_2

Ground State (Global minimum) E = -788.1376297 a.u.

C	2.998900	-0.873430	-0.513330
C	2.906165	0.458232	-0.897302
C	1.799763	1.222127	-0.530505
C	0.774583	0.666894	0.232934
C	0.846187	-0.691119	0.625020
C	1.970846	-1.434277	0.235139
H	3.858236	-1.467484	-0.800028
H	3.696732	0.911331	-1.484881
H	1.748943	2.260068	-0.839970
H	2.029482	-2.476808	0.529742
P	-0.749215	1.649307	0.591301
B	-0.242802	-1.386762	1.477682
H	-1.107264	-0.766446	2.012008
H	-0.176972	-2.560478	1.662144
H	-0.214679	2.911017	0.239515
H	-0.560751	1.827326	1.982382
C	-2.932227	-0.651424	-0.653359
O	-2.040129	-1.375277	-0.507782
O	-3.835721	0.046985	-0.811020

2nd Minimum (Activated State) E = -788.1244335 a.u.

C	-3.051738	0.530687	-0.319861
C	-2.906226	-0.857039	-0.367767
C	-1.667209	-1.423901	-0.112054
C	-0.605013	-0.570247	0.193695
C	-0.700383	0.830135	0.233887
C	-1.970600	1.355406	-0.029621
H	-4.022221	0.969035	-0.523855
H	-3.753010	-1.488558	-0.606173
H	-1.535133	-2.499912	-0.148025
H	-2.101910	2.431691	-0.012807
P	1.061210	-1.112661	0.516163
B	0.592622	1.723503	0.633087
H	0.945181	1.432625	1.761607
H	0.428314	2.897195	0.478971
H	1.309142	-2.397856	0.019440
H	1.342054	-1.192036	1.887768
C	2.263172	0.140602	-0.349069
O	1.801008	1.316259	-0.307123
O	3.240781	-0.330465	-0.850386

Transition State between the Global State and 2nd Minimum E = -788.1164995

C	-3.049906	0.635665	-0.357171
C	-2.943466	-0.745793	-0.484914
C	-1.733245	-1.371637	-0.207878
C	-0.645249	-0.603578	0.204920
C	-0.717238	0.796212	0.323991
C	-1.948792	1.391537	0.035255

H	-3.992165	1.124981	-0.575792
H	-3.796919	-1.334034	-0.800264
H	-1.644474	-2.448211	-0.307108
H	-2.039284	2.469529	0.114806
P	0.995625	-1.307593	0.524793
H	1.024778	-1.563961	1.909763
H	0.906461	-2.625613	0.037305
B	0.532521	1.656685	0.826515
H	1.041641	1.264499	1.842768
H	0.434309	2.839151	0.714789
C	2.475970	0.334324	-0.451386
O	1.801185	1.323053	-0.316819
O	3.428843	-0.200083	-0.847887

2P
E = -710.3354599 a.u.

C	-2.866868	0.051890	0.183472
C	-2.377232	-1.237947	-0.026654
C	-1.017579	-1.430106	-0.237417
C	-0.169403	-0.321890	-0.223231
C	-0.634353	0.985904	-0.024319
C	-2.007766	1.145785	0.184913
H	-3.928134	0.198901	0.350976
H	-3.052808	-2.085087	-0.024740
H	-0.626788	-2.429349	-0.406191
H	-2.407265	2.139972	0.357516
B	0.496058	2.127786	-0.086379
N	1.833587	1.218136	0.240811
H	1.923511	1.092759	1.249187
H	2.680262	1.675650	-0.087891
N	2.112489	-1.470337	0.686604
H	2.867962	-2.114340	0.516325
H	1.484747	-1.741492	1.431570
H	0.414300	2.996273	0.745434
H	0.655797	2.554078	-1.207190
P	1.621654	-0.421513	-0.541706

2P-CO_2
E = -898.8865202 a.u.

C	3.396500	0.250992	0.194006
C	3.012384	-1.082948	0.348925
C	1.673052	-1.420984	0.240987
C	0.748435	-0.403401	-0.018541
C	1.092212	0.952693	-0.176629
C	2.457670	1.243385	-0.062646
H	4.444760	0.514390	0.282246
H	3.753307	-1.846475	0.551417
H	1.352462	-2.452414	0.345475
H	2.779640	2.273519	-0.168943
P	-0.991125	-0.665530	-0.218932
B	-0.015192	2.085284	-0.551833

H	-0.404717	1.897958	-1.693419
H	0.404648	3.195751	-0.396995
N	-1.416039	-2.100944	0.493094
H	-1.021873	-2.251878	1.414654
H	-2.415806	-2.277486	0.492313
N	-1.602573	-0.806394	-1.758096
H	-1.640595	0.074057	-2.263079
H	-1.197494	-1.544105	-2.324079
C	-1.861727	0.828751	0.542599
O	-1.268315	1.944052	0.377584
O	-2.868231	0.570154	1.153212

3P
E = - -756.8196639 a.u.

C	1.661340	-2.506485	-0.172371
C	0.311216	-2.844453	-0.181920
C	-0.649034	-1.844001	-0.089091
C	-0.277307	-0.500027	-0.010827
C	1.083885	-0.147721	-0.013683
C	2.038893	-1.172798	-0.073918
H	2.416163	-3.282235	-0.231362
H	0.009034	-3.883142	-0.249490
H	-1.698506	-2.121296	-0.076646
H	3.095853	-0.922917	-0.051188
P	-1.508511	0.868353	0.189753
B	1.573989	1.352086	-0.016467
C	-2.582186	0.578983	-1.292583
H	-2.914423	-0.459632	-1.360972
H	-3.456999	1.229979	-1.235667
H	-2.025976	0.831750	-2.196486
C	-2.611852	0.150390	1.493097
H	-3.441009	0.839188	1.667136
H	-3.018287	-0.824427	1.215639
H	-2.050911	0.049913	2.423267
C	1.290118	2.272065	-1.256585
H	1.099884	3.309412	-0.967788
H	2.216442	2.287254	-1.850308
H	0.490231	1.924780	-1.912642
C	2.437830	1.878070	1.183042
H	3.050578	1.105758	1.654893
H	3.052916	2.749258	0.947567
H	1.715314	2.196490	1.949130

3P-CO_2
E = -945.4017562 a.u.

C	-3.312985	-0.898896	-0.150886
C	-2.640099	-2.121979	-0.103731
C	-1.259098	-2.123285	-0.032313
C	-0.575139	-0.899419	0.001336
C	-1.211300	0.352709	-0.050610
C	-2.614380	0.299250	-0.129755

H	-4.395502	-0.888153	-0.213701
H	-3.188688	-3.055403	-0.128903
H	-0.719074	-3.065248	-0.004413
H	-3.166358	1.230780	-0.182833
P	1.203835	-0.868276	0.083807
B	-0.461253	1.801890	0.074028
C	1.956491	-2.071738	-1.036686
H	1.749492	-3.088645	-0.701302
H	1.552901	-1.925007	-2.038484
H	3.032192	-1.890764	-1.048624
C	1.860979	-1.220952	1.735121
H	1.638238	-2.256833	1.996761
H	2.940727	-1.062518	1.718603
H	1.398212	-0.553977	2.460730
C	-0.191318	2.151531	1.634007
H	0.415467	1.402366	2.160371
H	0.327094	3.110801	1.732196
H	-1.135472	2.227854	2.183486
C	-1.215766	2.976587	-0.723583
H	-1.450822	2.711428	-1.760000
H	-2.152328	3.267186	-0.235970
H	-0.585695	3.870756	-0.748191
C	1.847246	0.819332	-0.488611
O	0.983587	1.733109	-0.618714
O	3.031485	0.817045	-0.724123

4P
E = -1352.3224296 a.u.

C	-3.036746	-1.982725	0.001513
C	-3.769627	-0.789207	0.000925
C	-3.124663	0.441215	-0.000034
C	-1.733130	0.420408	-0.000308
C	-0.976186	-0.761142	0.000356
C	-1.645598	-1.984969	0.001220
H	-3.572556	-2.925200	0.002227
H	-4.852393	-0.829512	0.001202
H	-3.689329	1.366468	-0.000539
H	-1.097021	-2.920003	0.001690
P	-0.335490	1.553274	-0.001915
B	0.554153	-0.244178	0.000061
C	-0.264890	2.634968	-1.461121
H	-1.062700	3.378053	-1.419845
H	0.703286	3.138375	-1.483516
H	-0.367433	2.022052	-2.355591
C	-0.263892	2.638956	1.454246
H	0.702663	3.145639	1.472264
H	-1.064293	3.379280	1.413508
H	-0.361696	2.028079	2.350652
C	1.391914	-0.506454	-1.350984
C	1.390853	-0.502535	1.352603
F	0.662999	-0.214160	-2.465206

F	2.512648	0.244626	-1.453025
F	1.777232	-1.789203	-1.491449
F	1.780304	-1.783773	1.495064
F	2.508970	0.252472	1.455329
F	0.659498	-0.211353	2.465496

4P-CO_2
E = -1540.9144876 a.u.

C	-0.200088	3.512263	0.167479
C	-1.591394	3.423734	0.203338
C	-2.189976	2.180438	0.104324
C	-1.384048	1.042198	-0.025593
C	0.017809	1.095250	-0.073280
C	0.583654	2.375097	0.032510
H	0.276863	4.482410	0.243819
H	-2.197677	4.315051	0.306601
H	-3.272309	2.096491	0.129574
H	1.661370	2.480485	0.012763
P	-2.149096	-0.562378	-0.167444
B	0.959153	-0.222219	-0.147783
C	-3.418948	-0.576245	-1.454184
H	-4.255568	0.060019	-1.162820
H	-2.986939	-0.210745	-2.385890
H	-3.758565	-1.604641	-1.586006
C	-2.919718	-1.157603	1.354239
H	-3.803941	-0.553006	1.563359
H	-3.208282	-2.199567	1.203569
H	-2.207093	-1.080368	2.172859
C	1.236478	-0.723950	1.377901
C	2.347430	0.044964	-0.946113
F	0.045803	-1.195935	1.905142
F	1.638075	0.243639	2.219915
F	2.102702	-1.736770	1.505772
F	3.017546	-1.079675	-1.243435
F	2.149864	0.687811	-2.124301
F	3.211665	0.822981	-0.243211
C	-0.874178	-1.814328	-0.754867
O	0.342135	-1.413122	-0.854423
O	-1.334663	-2.877970	-1.063168

5P
E = -788.9673718 a.u.

C	-2.600084	-1.483087	-0.163349
C	-3.021976	-0.175443	0.079968
C	-2.078790	0.825790	0.274522
C	-0.723024	0.499277	0.210748
C	-0.270587	-0.805903	-0.022508
C	-1.244595	-1.791352	-0.214130
H	-3.338172	-2.262801	-0.315753
H	-4.079335	0.058406	0.117939
H	-2.398255	1.845953	0.466854

H	-0.936794	-2.814179	-0.410194
P	0.608016	1.714978	0.485929
B	1.331607	-1.019150	-0.005385
N	1.805775	0.563974	-0.274266
H	1.833291	0.739476	-1.279734
H	2.744760	0.721073	0.087027
N	0.319179	2.797156	-0.780087
H	0.479311	3.780468	-0.631128
H	-0.352423	2.585653	-1.505974
C	1.918867	-1.929016	-1.202091
H	3.015500	-1.939372	-1.220612
H	1.607058	-2.971472	-1.074071
H	1.570320	-1.619020	-2.195056
C	1.880427	-1.418209	1.466053
H	1.520086	-2.415198	1.739410
H	2.976463	-1.459449	1.505479
H	1.543807	-0.738703	2.258999

5P-CO_2
E = -977.5178983 a.u.

C	-3.297666	-0.819649	-0.259111
C	-2.659509	-2.062925	-0.260543
C	-1.282585	-2.109067	-0.149964
C	-0.571863	-0.906747	-0.034353
C	-1.168804	0.367015	-0.047672
C	-2.570371	0.357503	-0.160965
H	-4.377381	-0.775865	-0.350243
H	-3.233155	-2.976627	-0.354460
H	-0.761621	-3.061050	-0.162524
H	-3.096330	1.304967	-0.186890
P	1.184719	-0.900398	0.162953
B	-0.372909	1.791703	0.114641
C	-0.103816	2.143618	1.676063
H	0.539059	1.427594	2.204237
H	0.380732	3.121201	1.765863
H	-1.047590	2.194166	2.229428
C	-1.092583	2.993661	-0.676793
H	-1.336535	2.740597	-1.713927
H	-2.018934	3.310709	-0.185905
H	-0.434184	3.867251	-0.697705
N	1.477506	-1.144599	1.787221
H	0.778732	-0.760696	2.413009
H	2.422929	-0.978439	2.113635
N	2.031275	-2.047007	-0.655746
H	2.794736	-1.696201	-1.223853
H	2.149799	-2.972701	-0.272409
C	1.847623	0.692467	-0.611780
O	1.065077	1.685380	-0.582587
O	2.956107	0.590686	-1.088085

6P

E = -1384.4790181 a.u.

C	2.131353	2.649312	0.082881
C	3.246216	1.823241	0.221709
C	3.086127	0.444424	0.267760
C	1.801891	-0.089917	0.158325
C	0.671014	0.722026	0.021014
C	0.854983	2.106316	-0.014337
H	2.262340	3.724648	0.051027
H	4.236694	2.255664	0.297692
H	3.949975	-0.202571	0.382943
H	-0.003516	2.759756	-0.121754
P	1.484258	-1.883085	0.256219
B	-0.712696	-0.073179	-0.046011
N	-0.258931	-1.586314	-0.352208
H	-0.252323	-1.740363	-1.363684
H	-0.906976	-2.266901	0.047477
N	2.242416	-2.398195	-1.147261
H	2.599311	-3.337477	-1.218431
H	2.507825	-1.771002	-1.894624
C	-1.689080	0.388616	-1.249325
C	-1.455124	-0.123803	1.391138
F	-2.520150	-0.967651	1.416872
F	-0.611750	-0.602075	2.348585
F	-1.895436	1.065818	1.825578
F	-2.835800	-0.325787	-1.332439
F	-2.045912	1.683564	-1.198834
F	-1.075661	0.208431	-2.456717

6P-CO_2

E = -1573.0302529 a.u.

C	-0.270999	3.498612	-0.016024
C	-1.661066	3.389083	0.038188
C	-2.239417	2.133849	0.013467
C	-1.413949	1.004975	-0.069110
C	-0.012239	1.077977	-0.131500
C	0.532853	2.370694	-0.095520
H	0.188979	4.479788	0.002879
H	-2.280733	4.275047	0.098616
H	-3.318632	2.028538	0.057819
H	1.608239	2.493904	-0.128269
P	-2.143053	-0.608469	-0.111674
B	0.949041	-0.226554	-0.131709
C	1.226544	-0.646494	1.419322
C	2.344041	0.020768	-0.926455
F	0.035882	-1.091455	1.978184
F	1.626197	0.364875	2.207736
F	2.091777	-1.650570	1.601317
F	3.032984	-1.108860	-1.154914
F	2.152375	0.597152	-2.138241
F	3.188796	0.846641	-0.254937
N	-2.756233	-0.925446	1.396756

H	-2.140005	-0.686860	2.167891
H	-3.182909	-1.837841	1.517709
N	-3.373092	-0.814340	-1.179207
H	-3.318157	-1.622713	-1.787052
H	-4.298438	-0.460773	-0.987398
C	-0.876340	-1.805873	-0.825983
O	0.357729	-1.456416	-0.789730
O	-1.342870	-2.807632	-1.300519

7P

E = -2368.4095704 a.u.

C	0.012302	-2.189016	3.658743
C	0.852631	-3.143055	3.088168
C	1.290099	-2.990372	1.779211
C	0.895901	-1.881087	1.021117
C	0.028880	-0.932848	1.588524
C	-0.406575	-1.100307	2.908861
H	-0.325362	-2.305450	4.681886
H	1.163051	-4.007068	3.664025
H	1.938410	-3.743299	1.349456
H	-1.079924	-0.373674	3.353618
P	1.336528	-1.512617	-0.734713
B	-0.494588	0.270707	0.716591
C	0.798193	-3.018735	-1.751080
C	3.228369	-1.371311	-0.703244
C	0.447542	1.505609	0.427631
C	0.563401	2.062118	-0.843597
C	1.253697	2.059141	1.412354
C	1.439956	3.089574	-1.133788
C	2.133416	3.103064	1.163502
C	2.228897	3.615390	-0.118149
C	-2.019596	0.326129	0.316301
C	-2.667268	1.503115	-0.082384
C	-2.845548	-0.805122	0.380552
C	-4.008429	1.558166	-0.420248
C	-4.188126	-0.785196	0.039292
C	-4.771942	0.403563	-0.365053
F	-2.016862	2.663768	-0.129889
F	-4.570837	2.702537	-0.786225
F	-6.052679	0.438959	-0.685672
F	-4.920891	-1.889871	0.104114
F	-2.372811	-1.984519	0.770109
F	-0.176637	1.584037	-1.844227
F	1.536969	3.582161	-2.363691
F	3.068920	4.605844	-0.378806
F	2.884627	3.606402	2.136643
F	1.203374	1.594338	2.663292
C	3.743278	-1.296107	-2.146170
H	4.788666	-0.971217	-2.135983
H	3.706553	-2.266983	-2.643859
H	3.173904	-0.577271	-2.742488

C	3.518212	-0.028747	-0.013099
H	3.095223	0.807862	-0.574376
H	3.116799	-0.010081	1.004463
H	4.601532	0.116658	0.051078
C	3.991461	-2.457971	0.063050
H	3.776275	-3.465697	-0.291991
H	5.065828	-2.284270	-0.064797
H	3.779294	-2.409748	1.132022
C	1.639830	-4.290617	-1.626003
H	1.684983	-4.655241	-0.598189
H	1.180312	-5.079255	-2.232249
H	2.656966	-4.148663	-1.994470
C	0.760758	-2.576035	-3.223992
H	1.753950	-2.374863	-3.626404
H	0.314542	-3.375150	-3.825108
H	0.153853	-1.675956	-3.348349
C	-0.642494	-3.323624	-1.321746
H	-1.277469	-2.439413	-1.425067
H	-1.050179	-4.105066	-1.970997
H	-0.701821	-3.667648	-0.287835

7P-CO_2

E = -2557.0169224 a.u.

C	0.564239	-0.175894	3.723098
C	1.955984	-0.051185	3.714364
C	2.617502	0.042724	2.505998
C	1.891147	0.023131	1.301316
C	0.495248	-0.075765	1.283359
C	-0.139413	-0.189310	2.534026
H	0.035419	-0.255411	4.665747
H	2.513493	-0.031801	4.642679
H	3.697064	0.134215	2.499554
H	-1.222289	-0.266828	2.562429
P	2.764762	0.068149	-0.271253
B	-0.471975	-0.040968	-0.017038
C	4.017860	-1.324326	-0.373966
C	3.419431	1.789723	-0.598731
C	-1.380313	1.323480	-0.094971
C	-2.392075	1.406228	-1.049335
C	-1.201747	2.474033	0.660339
C	-3.184255	2.525294	-1.235881
C	-1.972912	3.619328	0.506767
C	-2.972870	3.644775	-0.446277
C	-1.374290	-1.413745	0.006680
C	-2.662883	-1.512047	0.513458
C	-0.845683	-2.609232	-0.460133
C	-3.391885	-2.691625	0.532825
C	-1.533755	-3.811088	-0.464751
C	-2.824410	-3.851323	0.034093
F	-3.274308	-0.434978	1.032269
F	-4.625734	-2.721116	1.028145

F	-3.503937	-4.991805	0.046536
F	-0.965794	-4.922414	-0.927499
F	0.420123	-2.655250	-0.924266
F	-2.651687	0.356571	-1.835558
F	-4.142294	2.541238	-2.157577
F	-3.717508	4.731430	-0.610014
F	-1.748704	4.693538	1.261845
F	-0.237036	2.556730	1.595205
C	4.300056	1.821629	-1.854491
H	4.543287	2.867477	-2.062876
H	5.241197	1.287623	-1.715965
H	3.782237	1.412813	-2.723164
C	2.179242	2.675222	-0.814420
H	1.643887	2.417751	-1.730700
H	1.491540	2.619689	0.032440
H	2.516032	3.711709	-0.903020
C	4.189196	2.314208	0.621290
H	5.056863	1.702489	0.872572
H	4.551659	3.317263	0.379755
H	3.542777	2.396523	1.496714
C	5.348796	-0.925710	0.282247
H	5.238962	-0.644465	1.330231
H	6.002584	-1.801760	0.250693
H	5.853981	-0.120297	-0.251201
C	4.274719	-1.715402	-1.838614
H	4.600993	-0.875806	-2.451939
H	5.066854	-2.469600	-1.835918
H	3.388965	-2.146202	-2.302911
C	3.429151	-2.533261	0.370655
H	2.450492	-2.815376	-0.018521
H	4.108153	-3.377169	0.220356
H	3.338190	-2.352690	1.442466
C	1.492713	-0.122430	-1.659064
O	0.250696	-0.045044	-1.361025
O	1.958711	-0.187157	-2.767283

8P

E = -946.1472091 a.u.

C	-3.045578	-1.883420	-0.232873
C	-2.057867	-2.778498	0.180561
C	-0.792251	-2.304749	0.504137
C	-0.534157	-0.937836	0.396001
C	-1.511966	-0.014653	0.004386
C	-2.775085	-0.520866	-0.317165
H	-4.031243	-2.256491	-0.488643
H	-2.276160	-3.837802	0.248095
H	-0.013952	-2.992046	0.823518
H	-3.557332	0.160821	-0.638250
P	1.032444	-0.150283	0.873938
B	-1.067999	1.539910	0.051658
C	-1.341984	2.157385	1.530563

H	-0.838879	1.624993	2.347504
H	-1.074971	3.217971	1.609003
H	-2.415422	2.089581	1.735983
C	-1.683097	2.471338	-1.118860
H	-1.664045	2.026733	-2.119487
H	-2.735084	2.670676	-0.885775
H	-1.203464	3.456742	-1.181541
N	2.184138	-1.031494	0.013098
N	0.603585	1.359065	-0.147746
C	1.999481	-1.786046	-1.220000
H	2.546945	-1.325017	-2.051092
H	2.379650	-2.804567	-1.087200
H	0.945119	-1.845388	-1.482300
C	3.581265	-0.934810	0.415769
H	4.170539	-0.370700	-0.318293
H	3.657429	-0.435439	1.382132
H	4.016927	-1.935366	0.504371
C	0.928447	1.172037	-1.577434
H	0.705352	2.095682	-2.112148
H	1.987757	0.930935	-1.694963
H	0.314622	0.370621	-1.988027
C	1.362786	2.522893	0.357152
H	1.217060	2.617771	1.430810
H	2.429231	2.399313	0.145624
H	0.999244	3.426030	-0.137557

8P-CO_2
E = -1134.6959401 a.u.

C	2.918305	2.407442	0.045344
C	1.699229	3.089272	0.081437
C	0.523780	2.360445	0.045466
C	0.590600	0.960634	-0.011659
C	1.794913	0.237662	-0.074365
C	2.960222	1.022464	-0.039753
H	3.844287	2.971605	0.068148
H	1.674235	4.171004	0.129407
H	-0.437751	2.865430	0.062540
H	3.923065	0.526936	-0.094594
P	-0.906161	0.007565	-0.044107
B	1.882190	-1.395141	-0.136599
C	1.689360	-2.049913	1.337133
H	0.691301	-1.881419	1.761576
H	1.836093	-3.134048	1.292234
H	2.415780	-1.648381	2.052177
C	3.204705	-1.916487	-0.890798
H	3.368524	-1.432615	-1.859870
H	4.105527	-1.761284	-0.286730
H	3.128420	-2.993198	-1.069634
N	-1.438657	-0.285143	1.489172
N	-2.172563	0.706085	-0.851986
C	-0.782698	0.178843	2.707027

H	-1.500615	0.732978	3.319731
H	-0.406986	-0.673187	3.280514
H	0.058020	0.827747	2.467490
C	-2.474269	-1.300232	1.686759
H	-3.243090	-0.909611	2.359519
H	-2.936065	-1.561699	0.733617
H	-2.040066	-2.204692	2.125069
C	-3.108205	1.620646	-0.211054
H	-2.878157	2.664209	-0.455987
H	-4.120246	1.398507	-0.560165
H	-3.078305	1.494141	0.870877
C	-2.121715	0.814461	-2.308035
H	-1.453491	0.063027	-2.727020
H	-3.119738	0.633645	-2.713238
H	-1.779761	1.809001	-2.615341
C	-0.546671	-1.610619	-0.968449
O	0.677829	-1.947299	-1.023002
O	-1.512367	-2.168974	-1.433290

9P
E = -1541.6582025 a.u.

C	-0.197800	3.630155	-0.266889
C	-1.513397	3.496228	0.179268
C	-2.008641	2.242553	0.511868
C	-1.177367	1.129357	0.376995
C	0.147442	1.246000	-0.054267
C	0.627952	2.517493	-0.376738
H	0.183027	4.611667	-0.524301
H	-2.147436	4.370320	0.269052
H	-3.030345	2.130842	0.861986
H	1.653095	2.635892	-0.711516
P	-1.672285	-0.552923	0.863136
B	0.969438	-0.127440	-0.039530
C	1.753501	-0.253943	1.386260
C	2.059096	-0.262486	-1.242536
N	-3.045106	-0.810521	-0.066772
N	-0.184143	-1.262496	-0.132936
C	-3.435348	-0.157590	-1.312006
H	-3.426045	-0.869104	-2.145738
H	-4.450922	0.238101	-1.212571
H	-2.765262	0.667662	-1.543468
C	-3.927140	-1.916978	0.293311
H	-3.877261	-2.721263	-0.450501
H	-3.643251	-2.320320	1.265644
H	-4.961954	-1.566041	0.348223
C	-0.582774	-1.479908	-1.550100
H	0.232276	-1.973604	-2.078638
H	-1.470114	-2.113928	-1.580313
H	-0.788374	-0.521960	-2.024580
C	0.178628	-2.585761	0.448087
H	0.351154	-2.485819	1.516704

H	-0.645511	-3.283691	0.281110
H	1.081126	-2.956239	-0.038015
F	0.882230	-0.375612	2.430976
F	2.584299	-1.320770	1.489135
F	2.501910	0.827581	1.660151
F	3.163491	0.474034	-1.009462
F	2.494159	-1.536743	-1.446864
F	1.596434	0.150281	-2.451051

9P-CO_2
E = -1730.2099179 a.u.

C	-0.861691	3.498577	-0.711400
C	0.528216	3.582772	-0.808063
C	1.289717	2.455970	-0.557948
C	0.648442	1.254034	-0.221175
C	-0.746091	1.140312	-0.102447
C	-1.481937	2.306675	-0.366199
H	-1.465819	4.377439	-0.904886
H	1.005478	4.517552	-1.075253
H	2.371949	2.499183	-0.634074
H	-2.562684	2.277456	-0.302612
P	1.649502	-0.163926	0.169918
B	-1.486201	-0.263033	0.225183
C	-1.689288	-1.060653	-1.183547
C	-2.899877	-0.059115	0.996787
N	2.443773	-0.715531	-1.160875
N	2.828714	0.104205	1.304270
C	2.036072	-0.410338	-2.534684
H	2.929247	-0.159434	-3.112467
H	1.539354	-1.271964	-2.984652
H	1.349122	0.433246	-2.556540
C	3.240152	-1.942755	-1.034279
H	4.129867	-1.847365	-1.661409
H	3.542669	-2.094242	0.001352
H	2.658275	-2.810007	-1.359537
C	4.080497	0.769201	0.949207
H	4.025700	1.848782	1.131544
H	4.881806	0.353321	1.564460
H	4.319576	0.591220	-0.098744
C	2.425742	0.322420	2.695104
H	1.541550	-0.264140	2.943270
H	3.236873	-0.007668	3.346507
H	2.217344	1.380838	2.886117
F	-0.448623	-1.485520	-1.625025
F	-2.439631	-2.168382	-1.121904
F	-2.182753	-0.311993	-2.188047
F	-3.864418	0.456899	0.189319
F	-2.799911	0.801444	2.042078
F	-3.406499	-1.195985	1.500166
C	0.562277	-1.460086	1.001879
O	-0.700859	-1.207101	1.103167

O	1.143845	-2.420541	1.425555
